# Supplementary material for: Effect of goal-directed mobilisation intervention compared with standard care on physical activity among medical inpatients: protocol for the GoMob-in randomised controlled trial
Source: BMJ Open. 2022 May 11;12(5):e058509. doi: 10.1136/bmjopen-2021-058509 (PMC9109085; doi:10.1136/bmjopen-2021-058509)
Supplement: Supplementary data [file bmjopen-2021-058509supp001.pdf]

Klinik für Allgemeine Innere Medizin
Inselspital, 3010 Bern

Ziel-gerichtete Mobilisierung von Spitalpatienten (Goal-directed Mobilization of Medical inpatients – GoMob-in)

Diese Studie ist organisiert durch: Dr. med. Fabian D. Liechti, MD-PhD
Klinik für Allgemeine Innere Medizin KAIM
Inselspital / Universität Bern
Freiburgstrasse, 3010 Bern

Sehr geehrte Dame, sehr geehrter Herr,

Wir möchten Sie anfragen, ob Sie an einer klinischen Studie teilnehmen wollen. Im Folgenden wird Ihnen dieses Studienvorhaben dargestellt.

Detailliertere Information

1. Ziel der Studie

Wir möchten Sie hiermit bitten, an unserer klinischen Studie teilzunehmen. Die Studie untersucht, wie die Mobilität von Patienten im Spital verbessert werden könnte. Wir machen diese Studie, um herauszufinden, ob eine tägliche Zielvorgabe bei hospitalisierten Patienten Beweglichkeit, Lebensqualität und Überleben verbessert und Stürze, Verwirrtheit und Wiedereintritte ins Spital reduziert.

2. Auswahl

Es können alle Personen teilnehmen, die auf der Bettenstation der Allgemeinen Inneren Medizin des Inselspitals hospitalisiert werden (Eintritt vor weniger als zwei Tagen) und Physiotherapie durch die Stationsärztin oder den Stationsarzt verordnet erhalten haben. Ausserdem müssen Sie diese Einverständniserklärung verstehen und unterschreiben können. Nicht teilnehmen dürfen Personen welche voraussichtlich weniger als 5 Tage hospitalisiert werden, eine relevante Geheinschränkung (wie z. B. einen Beinbruch oder einen Schlaganfall mit Halbseitenlähmung) haben, vom Arzt Bettruhe verordnet bekommen oder schwanger/stillend sind.

3. Allgemeine Informationen

Viele Patienten, welche auf einer Bettenstation hospitalisiert werden, bewegen sich während des Spitalaufenthaltes wenig. Es ist bekannt, dass dieser Bewegungsmangel zu Komplikationen wie Muskelabbau, Stürzen, Verwirrtheit und verschlechterter Bewegungsfähigkeit führen kann. Deswegen wird empfohlen, dass sich die Patienten während der Hospitalisation viel bewegen – abhängig vom Gesundheitszustand und allenfalls durch den behandelnden Arzt verfügter Einschränkung.

Diese Studie wird an der Klinik für Allgemeine Innere Medizin am Inselspital Bern durchgeführt. Wir planen 160 Personen in die Studie einzuschliessen. In dieser Studie wollen wir klären, ob eine klare Kommunikation und Angabe eines individuellen Tagesziels die Beweglichkeit von hospitalisierten Personen verbessern kann.

Die Teilnehmenden werden zufällig einer von 2 Gruppen zugeordnet: einer Motivations-Gruppe und einer Kontroll-Gruppe. Die Wahrscheinlichkeit der Zuteilung auf eine Gruppe beträgt 50%. Zur Messung der Bewegung, wird Ihnen während des Spitalaufenthaltes ein Schrittzähler angehängt. Dieser misst Ihre Bewegung, woraus ein Computerprogramm berechnen kann, ob sie schlafen, stehen, sitzen oder gehen. In der Motivations-Gruppe wird den Patienten jeden Tag vorgegeben,

wie viel sie sich mindestens bewegen sollen. In der Kontroll-Gruppe bewegen sich die Patienten unabhängig von einem Tagesziel.

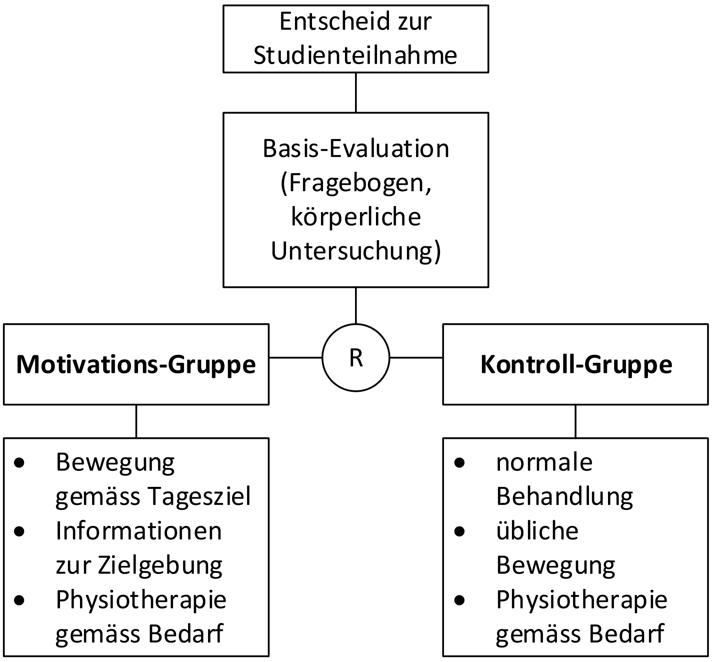

Abbildung 1: Übersicht der beiden Gruppen. (R = zufällige Zuordnung zu einer der beiden Gruppen)

Die aktive Teilnahme dauert solange wie der Spitalaufenthalt, jedoch höchstens 14 Tage und wird mit einem Fragebogen welcher nach 3 Monaten telefonisch beantwortet wird, abgeschlossen.

Wir machen diese Studie so, wie es die Gesetze in der Schweiz vorschreiben. Ausserdem beachten wir alle international anerkannten Richtlinien. Die zuständige Kantonale Ethikkommission hat die Studie geprüft und bewilligt.

Eine Beschreibung dieser Studie finden Sie auch auf der Internetseite des Bundesamtes für Gesundheit: www.kofam.ch

4. Ablauf

Nach Einwilligung zur Teilnahme an der Studie wird eine Eintrittsbefragung und -Untersuchung durchgeführt. Diese beinhaltet folgendes:

- Überprüfung der Ein- und Ausschlusskriterien der Studie
- Fragen zu Ihrer Person wie Grösse, Gewicht, Wohnort, Gehhilfen, Vorerkrankungen, etc.
- Fragebogen zur Gangunsicherheit, Selbständigkeit und Lebensqualität (ca. 30 min)
- Körperliche Untersuchung Ihrer Beweglichkeit (ca. 10-15 min)

Für die Studie sind keine zusätzlichen Blutentnahmen oder Röntgenuntersuchungen notwendig. Bei Frauen im gebährfähigen Alter wird aus einer bereits vorhandenen Blutprobe das Schwangerschaftshormon analysiert um eine Schwangerschaft auszuschliessen.

Die Untersuchung der Beweglichkeit wird am fünften Tag und vor Spitalaustritt oder spätestens nach 14 Tagen wiederholt. Sie müssen also für die Studie nicht länger im Spital bleiben als sonst vorgesehen.
Angaben zu ihrer Gesundheit wie z. B. Diagnosen, eingenommene Medikamente, Stürze werden wir zudem nach Ihrem Austritt aus Ihrer elektronischen Krankenakte entnehmen.

Klinik für Allgemeine Innere Medizin
Inselspital, 3010 Bern

Wir werden ihnen ein Sturztagebuch mit nach Hause geben, wo sie allfällige Stürze dokumentieren können. Nach 3 Monaten werden wir Sie telefonisch kontaktieren um einige Fragen zu Ihrer Gesundheit zu stellen (Fragebogen zur Lebensqualität, zur Gangunsicherheit, zur Selbständigkeit, zum aktuellen Aufenthaltsort u. ä.). Sollten wir Sie wiederholt nicht erreichen, werden wir Ihre Angehörigen oder Ihren Hausarzt kontaktieren, um diese Fragen klären zu können.

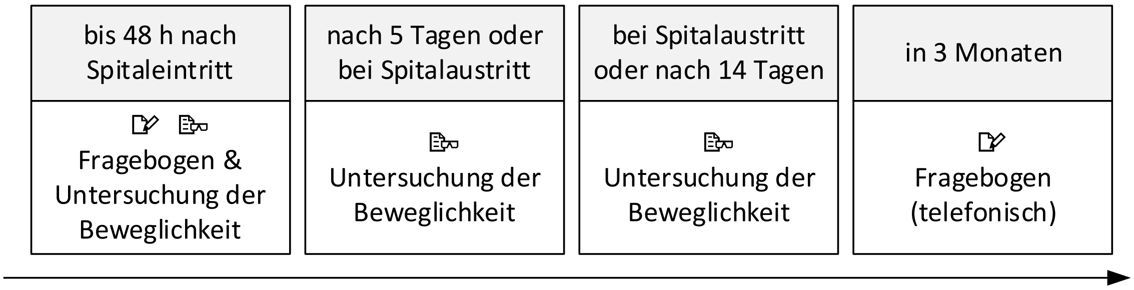

Abbildung 2: Ablauf der Untersuchungen während der Studie

Es kann sein, dass wir Sie von der Studie vorzeitig ausschliessen müssen. Das kann geschehen, wenn die Studie vorzeitig abgebrochen wird oder wir bei Ihnen ein Ausschlusskriterium finden, welches bei Einschluss nicht bekannt war.

5. Nutzen
Die Resultate der Studie können wichtig sein zur Erforschung der Mobilität bei Spitalpatienten.

6. Rechte
Sie nehmen freiwillig teil. Wenn Sie nicht mitmachen oder später Ihre Teilnahme zurückziehen wollen, müssen Sie dies nicht begründen. Ihre medizinische Behandlung/Betreuung ist unabhängig von Ihrem Entscheid gewährleistet. Sie dürfen jederzeit Fragen zur Studienteilnahme stellen. Wenden Sie sich dazu bitte an die Person, die am Ende dieser Information genannt ist.

7. Pflichten
Als Teilnehmer ist es notwendig, dass Sie

- sich an die notwendigen Vorgaben und Anforderungen der Studie durch den Prüfplan halten,
- Ihren Prüfarzt über den Verlauf der Erkrankung informieren und neue Symptome, neue Beschwerden und Änderungen im Befinden melden.

8. Risiken und Belastungen für die Teilnehmenden
Es sind keine Risiken der Studienintervention zu erwarten.

9. Andere Behandlungsmöglichkeiten
Sie müssen bei dieser Studie nicht teilnehmen. Wenn Sie nicht mitmachen, können Sie z. B. selbst auf eine ausreichende Bewegung während des Spitalaufenthalts achten.

10. Ergebnisse aus der Studie
Der Prüfarzt wird Sie während der Studie über alle neuen Erkenntnisse informieren, die den Nutzen der Studie oder Ihre Sicherheit und somit Ihr Einwilligung zur Teilnahme an der Studie beeinflussen können. Sie werden die Information mündlich und schriftlich erhalten.

11. Vertraulichkeit der Daten
Für diese Studie werden Ihre persönlichen und medizinischen Daten erfasst. Nur sehr wenige Fachpersonen werden Ihre unverschlüsselten Daten sehen, und zwar ausschliesslich, um Aufgaben im Rahmen der Studie zu erfüllen. Bei der Datenerhebung zu Studienzwecken werden die Daten verschlüsselt. Verschlüsselung bedeutet, dass alle Bezugsdaten, die Sie identifizieren

könnten (Name, Geburtsdatum), gelöscht und durch einen Schlüssel ersetzt werden. Die Schlüssel-Liste bleibt immer im Inselspital. Diejenigen Personen, die den Schlüssel nicht kennen, können daher keine Rückschlüsse auf Ihre Person ziehen. Bei einer Publikation sind die zusammengefassten Daten daher auch nicht auf Sie als Einzelperson rückverfolgbar. Ihr Name taucht niemals im Internet oder einer Publikation auf. Manchmal gibt es die Vorgabe zur Publikation bei einer Zeitschrift, dass Einzel-Daten (sogenannte Roh-Daten) übermittelt werden müssen. Wenn Einzel-Daten übermittelt werden müssen, dann sind die Daten immer verschlüsselt und somit ebenfalls nicht zu Ihnen als Person rückverfolgbar. Alle Personen, die im Rahmen der Studie Einsicht in Ihre Daten haben, unterliegen der Schweigepflicht. Die Vorgaben des Datenschutzes werden eingehalten und Sie als teilnehmende Person haben jederzeit das Recht auf Einsicht in Ihre Daten.
Möglicherweise wird diese Studie durch die zuständige Ethikkommission überprüft. Der Prüfarzt muss eventuell Ihre persönlichen und medizinischen Daten für solche Kontrollen offenlegen. Alle Personen müssen absolute Vertraulichkeit wahren.
Es ist möglich, dass Ihr nachbehandelnder Arzt kontaktiert wird, um Auskunft über Ihren Gesundheitszustand zu geben.

12. Rücktritt
Sie können jederzeit aufhören und von der Studie zurücktreten, wenn Sie das wünschen. Die bis dahin erhobenen Daten werden noch verschlüsselt ausgewertet, weil das ganze Projekt sonst seinen Wert verliert.
Nach der Auswertung werden Ihre Daten vollständig anonymisiert, d.h. Ihre Schlüsselzuordnung wird vernichtet, so dass danach niemand mehr erfahren kann, dass die Daten ursprünglich von Ihnen stammten.

13. Entschädigung für Teilnehmende
Wenn Sie an dieser Studie teilnehmen, bekommen Sie dafür keine Entschädigung. Es entstehen Ihnen oder Ihrer Krankenkasse keine Kosten durch die Teilnahme.

14. Haftung
Die Institution, die die Studie veranlasst hat und für die Durchführung verantwortlich ist, haftet für Schäden, welche Ihnen im Zusammenhang mit den Forschungshandlungen (z.B. Untersuchungen) entstehen könnten. Die Voraussetzungen und das Vorgehen dazu sind gesetzlich geregelt.
Falls Sie einen Schaden erlitten haben, so wenden Sie sich bitte an den Prüfarzt.

15. Finanzierung der Studie
Die Studie wird grösstenteils von der SGAIM Foundation (Stiftung der Schweizerischen Gesellschaft für Allgemeine Innere Medizin) bezahlt.

16. Kontaktperson(en)
Bei Fragen, Unsicherheiten oder Notfällen, die während der Studie oder danach auftreten, können Sie sich jederzeit an eine dieser Kontaktpersonen wenden.

Prüfarzt: Dr. med. Fabian D. Liechti, MD-PhD
Klinik für Allgemeine Innere Medizin KAIM
Inselspital / Universität Bern
Freiburgstrasse, 3010 Bern
fabian.liechti@insel.ch
Tel. +41 31 632 21 11 / Sucher: 181-7253

Klinik für Allgemeine Innere Medizin
Inselspital, 3010 Bern

Einwilligungserklärung

Schriftliche Einwilligungserklärung zur Teilnahme an einem Studienprojekt
Bitte lesen Sie dieses Formular sorgfältig durch. Bitte fragen Sie, wenn Sie etwas nicht verstehen oder wissen möchten. Für die Teilnahme ist Ihre schriftliche Einwilligung notwendig.

**BASEC-Nummer (nach Einreichung):**	BASEC 2020-02305
**Titel der Studie (wissenschaftlich und Laiensprache)**:	Ziel-gerichtete Mobilisierung von Spitalpatienten (Goal-directed Mobilization of Medical inpatients – GoMob-in)
**verantwortliche Institution (Sponsor mit Adresse)**:	Inselspital, Klinik für Allgemeine Innere Medizin, Freiburgstrasse, 3010 Bern
**Ort der Durchführung**:	Bern
**Verantwortlicher Prüfarzt / Studienkoordinator am Studienort:** Name und Vorname in Druckbuchstaben:	Dr. med. Fabian D. Liechti
**Teilnehmerin/Teilnehmer:** Name und Vorname in Druckbuchstaben: Geburtsdatum:	☐ weiblich ☐ männlich

- Ich wurde vom unterzeichnenden Prüfarzt mündlich und schriftlich über den Zweck, den Ablauf der Studie, über mögliche Vor- und Nachteile sowie über eventuelle Risiken informiert.
- Ich nehme an dieser Studie freiwillig teil und akzeptiere den Inhalt der abgegebenen schriftlichen Information. Ich hatte genügend Zeit, meine Entscheidung zu treffen.
- Ich wurde über mögliche andere Behandlungen und Behandlungsverfahren aufgeklärt.
- Meine Fragen im Zusammenhang mit der Teilnahme an dieser Studie sind mir beantwortet worden. Ich behalte die schriftliche Information und erhalte eine Kopie meiner schriftlichen Einwilligungserklärung.
- Ich bin mir bewusst, dass die in der Teilnehmerinformation genannten Pflichten einzuhalten sind. Im Interesse meiner Gesundheit kann mich der Prüfarzt jederzeit von der Studie ausschliessen.
- Ich bin einverstanden, dass mein Hausarzt über meine Teilnahme an der Studie informiert wird.
- Ich bin einverstanden, dass die zuständigen Fachleute des Sponsors, der zuständigen Ethikkommission zu Prüf- und Kontrollzwecken in meine unverschlüsselten Daten Einsicht nehmen dürfen, jedoch unter strikter Einhaltung der Vertraulichkeit.
- Bei Studienergebnissen, die direkt meine Gesundheit betreffen, werde ich informiert. Wenn ich das nicht wünsche, informiere ich meinen Prüfarzt.
- Ich weiss, dass meine gesundheitsbezogenen und persönlichen Daten nur in verschlüsselter Form zu Forschungszwecken für diese Studie weitergegeben werden können.
- Ich kann jederzeit und ohne Angabe von Gründen von der Studienteilnahme zurücktreten. Meine weitere medizinische Behandlung ist unabhängig von der Studienteilnahme immer gewährleistet. Die bis zum Rücktritt erhobenen Daten werden für die Auswertung zur Studie verwendet.
- Die Haftpflichtversicherung des Spitals/der Institution kommt für allfällige Schäden auf.

Liechti FD, *et al. BMJ Open* 2022; 12:e058509. doi: 10.1136/bmjopen-2021-058509

Klinik für Allgemeine Innere Medizin
Inselspital, 3010 Bern

Ort, Datum	Unterschrift Teilnehmerin/Teilnehmer

Bestätigung des Prüfarztes/der Prüfperson: Hiermit bestätige ich, dass ich dieser Teilnehmerin/ diesem Teilnehmer Wesen, Bedeutung und Tragweite der Studie erläutert habe. Ich versichere, alle im Zusammenhang mit dieser Studie stehenden Verpflichtungen gemäss dem geltenden Recht zu erfüllen. Sollte ich zu irgendeinem Zeitpunkt während der Durchführung der Studie von Aspekten erfahren, welche die Bereitschaft der Teilnehmerin/ des Teilnehmers zur Teilnahme an der Studie beeinflussen könnten, werde ich sie/ ihn umgehend darüber informieren.

Ort, Datum	Name und Vorname der Prüfärztin / des Prüfarztes / des Studienkoordinators / der Studienkoordinatorin in Druckbuchstaben<br><br>Unterschrift der Prüfärztin/des Prüfarztes des Studienkoordinators / der Studienkoordinatorin

Liechti FD, *et al. BMJ Open* 2022; 12:e058509. doi: 10.1136/bmjopen-2021-058509
