# Supplementary material for: Effect of goal-directed mobilisation intervention compared with standard care on physical activity among medical inpatients: protocol for the GoMob-in randomised controlled trial
Source: BMJ Open. 2022 May 11;12(5):e058509. doi: 10.1136/bmjopen-2021-058509 (PMC9109085; doi:10.1136/bmjopen-2021-058509)
Supplement: Supplementary data [file bmjopen-2021-058509supp002.pdf]

Stufe	**Tages-Ziel** (mindestens 3 x/Tag)
**8**	**≥ 75 m gehen (30 min oder Treppe) & keine Bettruhe**
**7**	**≥ 75 m gehen**
**6**	**≥ 7,5 m gehen**
**5**	**≥ 10 Schritte gehen**
**4**	**≥ 1 Minute stehen**
**3**	**Transfer auf den Stuhl / Nachtstuhl**
**2**	**An die Bettkante sitzen**
**1**	**Aktivitäten im Bett / unselbständiger Transfer**

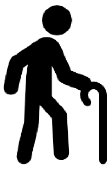
mit Gehstock

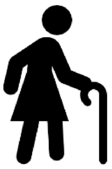
mit Gehstock

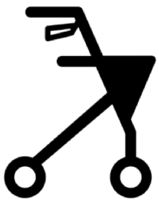
mit Rollator

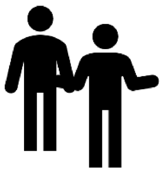
in Begleitung
