# Supplementary material for: Effect of goal-directed mobilisation intervention compared with standard care on physical activity among medical inpatients: protocol for the GoMob-in randomised controlled trial
Source: BMJ Open. 2022 May 11;12(5):e058509. doi: 10.1136/bmjopen-2021-058509 (PMC9109085; doi:10.1136/bmjopen-2021-058509)
Supplement: Author's manuscript [file bmjopen-2021-058509.draft_revisions.pdf]

BMJ Open is committed to open peer review. As part of this commitment we make the peer review history of every article we publish publicly available.

When an article is published we post the peer reviewers' comments and the authors' responses online. We also post the versions of the paper that were used during peer review. These are the versions that the peer review comments apply to.

The versions of the paper that follow are the versions that were submitted during the peer review process. They are not the versions of record or the final published versions. They should not be cited or distributed as the published version of this manuscript.

BMJ Open is an open access journal and the full, final, typeset and author-corrected version of record of the manuscript is available on our site with no access controls, subscription charges or pay-per-view fees (http://bmjopen.bmj.com).

If you have any questions on BMJ Open's open peer review process please email info.bmjopen@bmj.com

BMJ Open

Goal-directed mobilization of medical inpatients (GoMob-in) – Protocol for a randomized, controlled trial

Journal:	*BMJ Open*
Manuscript ID	bmjopen-2021-058509
Article Type:	Protocol
Date Submitted by the Author:	04-Feb-2022
Complete List of Authors:	Liechti, Fabian; University of Bern, Department of General Internal Medicine<br>Heinzmann, Jeannelle; University of Bern, Department of General Internal Medicine<br>Schmidt Leuenberger, Joachim; Inselspital University Hospital Bern, Department of Physiotherapy<br>Limacher, Andreas; Universitat Bern Institut fur Sozial- und Praventivmedizin<br>Wertli, Maria M; Inselspital Universitatsspital Bern, Department of General Internal Medicine<br>Verra, Martin; Inselspital University Hospital Bern, Department of Physiotherapy
Keywords:	Quality in health care < HEALTH SERVICES ADMINISTRATION & MANAGEMENT, INTERNAL MEDICINE, Rehabilitation medicine < INTERNAL MEDICINE, PREVENTIVE MEDICINE

SCHOLARONE™
Manuscripts

Goal-directed mobilization of medical inpatients (GoMob-in) – Protocol for a randomized, controlled trial

Fabian D. Liechti[1], Jeannelle Heinzmann[1], Joachim M. Schmidt Leuenberger[2], Andreas Limacher[3], Maria M. Wertli[1], Martin L. Verra[2]

[1]Department of General Internal Medicine, Inselspital, Bern University Hospital, University of Bern, Bern, Switzerland

[2]Department of Physiotherapy, Insel Group, Bern University Hospital, Bern, Switzerland.

[3]CTU Bern, University of Bern, Bern, Switzerland

Corresponding author:

Fabian Liechti, MD, PhD

Department of General Internal Medicine

Bern University Hospital

Freiburgstrasse 8

3010 Bern

Mail: fabian.liechti@insel.ch

Phone: +41 31 632 21 11

For peer review only

For peer review only - http://bmjopen.bmj.com/site/about/guidelines.xhtml

Abstract

Introduction: Despite the fact that immobilization is a major contributor to morbidity and mortality, patients hospitalized in general internal medicine (GIM) wards spend up to 50% of time in bed. Previous studies in selected patient populations such as intensive care patients, showed increased mobility after implementation of goal-directed mobilization (GDM). Due to the study design used so far, the degree of evidence is generally low. The effect of GDM on short and long-term clinical outcomes and economically relevant indicators in patients hospitalized in acute internal medicine wards is currently unknown.

Objectives: To evaluate the effect of GDM compared to standard care in acutely hospitalized patients in GIM on physical activity as assessed by the de Morton Mobility Index (DEMMI) score.

Methods and analysis: GoMob-in is a randomized, controlled, open-label study with blinded outcome assessment. We plan to enroll 160 inpatients with indication for physiotherapy on GIM wards of a tertiary hospital. Adult patients newly hospitalized on general internal medicine wards will be included in the study. The primary outcome will be the change in the DEMMI score between baseline and 5 days follow-up. Secondary outcomes include number of falls, delirium episodes, length of stay, and – at three months follow-up – independence during activities of daily living, quality of life, number of rehospitalizations, and all-cause mortality. Patients in the intervention group will be attributed a regularly updated individual mobility goal level made visible for all stakeholders and get a short educational intervention on GDM.

Ethics and Dissemination: This study has been approved by the responsible Ethics Board (Ethikkommission Bern / 2020-02305). Results will be published in open access policy peer-reviewed journals. The study is funded by SGAIM Foundation, Bern, Switzerland and Fondation Sana, Bern, Switzerland.

Trial registration: clinicaltrials.gov (NCT04760392)

For peer review only

Article Summary

Strengths and limitations of this study

- This is the first study evaluating goal-directed mobilisation in hospitalised general internal medicine patients using blinded outcome assessment.
- The primary endpoint physical activity will be assessed by change in the de Morton Mobility Index (DEMMI) score between baseline and day 5, a reliable tool without floor or ceiling effects.
- Activity will be recorded by an accelerometer to record the effect of the intervention.
- The study will also assess independence during activities of daily living, quality of life, number of rehospitalizations, and all-cause mortality at 3 months follow-up.
- The study design is limited by the fact that blinding is not feasible for the interventions and some outcomes.

Keywords: mobilisation, goal-directed mobility, randomized controlled trial, physiotherapy, interprofessional, outcome

Word count: 3336 words.

Protocol Version 1.5, 27.08.2021

For peer review only

Introduction

Decreased mobility during hospitalization is associated with muscle loss, risk of falls, delirium, functional decline, and an increased mortality, especially in the elderly.[1-4] Bedrest of 10 days in healthy elderly resulted in a substantial loss of muscle strength, power, aerobic capacity, and a reduction in physical activity.[5] Evidence suggests that interventions that promote early mobility have the potential to decrease the risk of falls,[6 7] to reduce functional decline during hospitalization,[8] to shorten duration of delirium[9] and to reduce the median LOS and mortality.[9 10] Therefore, early mobilization is recommended in most patients to prevent functional decline and frailty.[11 12] Despite recommendations to mobilize patients early, a low level of physical activity is still common and many patients spend most of the time in bed during hospitalization, irrespective of patient age.[3 12-14] In a recent Dutch study, inpatients spent almost half of the time in bed and were active only 10% of the time.[15] Most studies assessing interventions on mobilization focused on selected patients such as geriatric patients or those admitted to intensive care units. Further, most studies failed to monitor mobility or long-term functional outcomes or had a retrospective design.[2 12 16-18]

Implementation of early mobilization in general internal medicine (GIM) remains challenging and staff resources-demanding, because mobilization is usually supervised by health care professionals such as physiotherapists. Individual goal-achievement strategies have the potential to complement physiotherapy in rehabilitation.[12 19] Thereby, a mobility goal is generally defined depending on the individual potential of the patient and communicated to the interprofessional team caring for the patient. Goal-directed mobility (GDM) programs in GIM showed in temporal cohorts a reduction in length-of hospital-stay (LOS) and a decrease of falls.[20 21] Most evidence stems from studies which usually integrated several other organizational changes or were non-randomized trials, e.g., implementation of a mobility scale and corresponding mobility goal in hospital wards.[22] However, pre-post settings do not consider general trends, e.g. reduction in LOS and comparators may differ significantly. In one randomized controlled trial, a mobility program using goal-setting, reduced the decline in

For peer review only

activities of daily living during acute hospitalization.[7 23] However, it included only a small number of older veterans, was labor intense and lacked long-term follow-up.[7]

Due to the low number of studies of limited methodological quality, the level of evidence is low and the impact of GDM on clinical and economic outcomes in general internal medicine patients is widely unknown.[24]

Therefore, this study aims to evaluate a standardized GDM program in acutely hospitalized medical inpatients on functional capacity during hospitalization and up to 3 months after study inclusion. All patients will receive physiotherapy treatment as indicated by the physician in charge. Compared to a control group with standard care, patients in the intervention group will receive additional instructions for GDM, physiotherapists will work with goal-setting, and the goal will be indicated for all stakeholders at the bedside.

Methods and analysis

The methods reporting of this randomized trial will follow the recommendations of the SPIRIT statement (**Table 1**).[25]

Table 1: Trial registration data.

**Data category**	**Information**
Primary registry and trial identifying number	ClinicalTrials.gov<br>NCT04760392
Date of registration in primary registry	February 18, 2021
Secondary numbers	SNCTP000004280, BASEC2020-02305
Source(s) of monetary or material support	Swiss Society of General Internal Medicine (SGAIM) Foundation, Bern, Switzerland<br>Fondation Sana, Bern, Switzerland
Primary sponsor	Swiss Society of General Internal Medicine (SGAIM) Foundation, Bern, Switzerland (grant number: not applicable)

For peer review only - http://bmjopen.bmj.com/site/about/guidelines.xhtml

For peer review only

Secondary sponsor	Fondation Sana, Bern, Switzerland (grant number GF 2021-0048)
Contact for public queries	FDL fabian.liechti@insel.ch
Contact for scientific queries	FDL<br>Department of General Internal Medicine, Inselspital, Bern University Hospital, Bern, Switzerland
Public title	Goal-directed mobilization of medical inpatients – a randomized, controlled trial (GoMob-in trial)
Scientific title	Goal-directed mobilization of medical inpatients – a randomized, controlled trial (GoMob-in trial)
Countries of recruitment	Switzerland
Health condition(s) or problem(s) studied	Inpatient mobility
Intervention(s)	Intervention group: Goal-directed mobilisation
	Control group: Standard of care
Key inclusion and exclusion criteria	Ages eligible for study: 18 years or older<br>Sexes eligible for study: both<br>Accepts healthy volunteers: no
	Inclusion criteria: Acute hospitalization on the general internal medicine ward, indication for physiotherapy
	Exclusion criteria: Inability to follow study procedures and give informed consent themselves, expected hospital stay for <5 days, bedrest, injuries or relevant neurologic deficits one or both lower extremities directly impairing walking capacity, terminal illness
Study type	Interventional<br>Allocation: randomized intervention<br>Masking: single blind (primary outcome assessor)<br>Primary purpose: prevention
Date of first enrolement	14.09.2021
Target sample size	160

For peer review only - http://bmjopen.bmj.com/site/about/guidelines.xhtml

Recruitment status	Recruiting
Primary outcome	Change of the de Morton Mobility Index (DEMMI) score between baseline and day 5
Key secondary outcome	Change in DEMMI score between baseline and discharge.<br>Mobilization time measured by accelerometer between inclusion and day 5.<br>Mobilization time between inclusion and discharge.<br>Number of in-hospital falls.<br>Number of delirium episodes.<br>Length-of-hospital-stay.<br>Change in Falls Efficacy Scale - International (FES-I) between baseline and 3 months.<br>Number of re-hospitalizations and all-cause mortality until 3 months.

Study setting

This is an investigator initiated trial. The funder has no role in the study design, the trial oversight, data collection, analysis of the study, and publication of the results. The Goal-directed Mobilization of Medical Inpatients (GoMob-in) trial is a pragmatic, randomized, controlled, open-label study with a 3-month follow-up and a blind outcome assessment, conducted at the Department of General Internal Medicine, Inselspital, Bern University Hospital, Bern, Switzerland.

Objectives

To evaluate the effect of GDM compared to standard care in hospitalized patients in GIM on physical activity as assessed by the de Morton Mobility Index (DEMMI) score.

Hypothesis

GDM improves physical activity assessed by DEMMI compared to standard of care in hospitalized patients in GIM.

Eligibility criteria

<u>Inclusion criteria:</u>

Subjects fulfilling all of the following inclusion criteria are eligible for the study:

- Acute hospitalization on the GIM ward (elective or urgent admission, at latest on the second day after internal or external hospital admission)

For peer review only - http://bmjopen.bmj.com/site/about/guidelines.xhtml

- Age 18 years or older
- Indication for physiotherapy
- Informed consent as documented by signature (written informed consent)

Exclusion criteria:

The presence of any one of the following exclusion criteria will lead to exclusion of the subjects:

- Inability to follow study procedures, i.e., due to language problems (unable to read, speak or understand German), psychological disorders, severe dementia (defined as to levels 5 - 7 in the Global Deterioration Scale),[26] blindness, patients unable to provide informed consent themselves
- Expected hospital stay for <5 days
- Medically indicated bedrest for more than 24 h, e.g. after surgery
- Injuries or relevant neurologic deficits one or both lower extremities directly impairing walking capacity (e.g. fractures, hemiplegia, previous use of a wheelchair or bedriddenness)
- Terminal illness
- Pregnancy or breast feeding
- Previous enrolment in this study
- A study participant in the same patient room
- Enrolment of the investigator, his/her family members, employees and other dependent persons

Estimated sample size and power

The sample size calculation was based on the primary outcome of a clinically meaningful change in the de Morton Mobility Index (DEMMI) score between baseline and 5 days follow-up. The DEMMI score ranges from 0 to 100 points with 0 indicating no physical activity and 100 indicating maximal physical activity. Based on previous studies on changes in DEMMI scores during short-term hospitalization of roughly 10 days,[27 28] we expect a difference in change between the two groups of 5 score points. In an observational study of participants with low

For peer review only - http://bmjopen.bmj.com/site/about/guidelines.xhtml

and high activity, standard deviation (SD) for changes in the DEMMI score was 7.8 and 8.5 points, respectively (personal communication).[27] In a study on geriatric inpatients the SD was between 12.5 and 15.4 points for absolute DEMMI scores at admission and discharge, respectively.[28] The SD for changes is typically smaller than for absolute values; we therefore expect a rather conservative an SD of about 10 points for the change in our study.

To detect a difference in change of 5 score points with a SD of 10 points, at a two-sided alpha level of 0.05 with a power of 0.8, a sample size of 2x 64 participants is required. Based on an estimated drop-out rate of 20%, we aim to include 160 patients into the study (80 in each group).

Primary outcome

The primary outcome will be the change in physical activity level measured by performance in the DEMMI score between baseline and 5 days follow-up.[29] Trained study personnel will evaluate physical activity by using DEMMI score at baseline before randomization, while a physiotherapist blinded to the group allocation will assess DEMMI score 5 days later.

Secondary outcome

- Change in DEMMI score between baseline at study inclusion (at latest on the second day after hospital admission) and discharge. Patients hospitalized for more than 2 weeks will be assessed on day 14 after study inclusion instead of at hospital discharge.
- Percentage of time moving between study inclusion and day 5. Activity will be measured using an accelerometer (GENEActiv, Activinsights, Kimbolton, Cambridgeshire, United Kingdom) validated to assess activity in the hospital setting.[13 14 30]
- Percentage of mobilization time between baseline and discharge or at 14 days (whichever occurs first).
- Number of in-hospital falls and number of in-hospital delirium episodes.
- Discharge destination (death, acute care hospital, rehabilitation, home, nursing home)
- LOS defined as days between hospital admission and discharge.

For peer review only - http://bmjopen.bmj.com/site/about/guidelines.xhtml

- Change in independence during activities of daily living (Barthel index) between baseline and 3 months after study inclusion.[31 32]
- Change in Falls Efficacy Scale - International (FES-I) between baseline and 3 months after study inclusion.[33 34]
- Change in quality of life (EQ-5D-5L) (visual analogue scale [EQ-VAS] and mobility dimension) between baseline and 3 months after study inclusion.[35-37]
- Total number of falls, number of rehospitalizations and all-cause mortality within 3 months after study inclusion.
- Reaching the minimal clinically important difference (MCID) of a change in DEMMI score (9 points) between baseline and discharge.[29 38]

Study procedure

This study will give evidence about a specific, easily implementable intervention using little resources to promote mobility and evaluate its impact on significant clinical outcomes of GIM inpatients (**Figure 1**).

Pre-screening and recruitment:

All patients hospitalised on the GIM department will be pre-screened during weekdays within the first two days of hospitalisation for study participation.

Baseline assessment:

Study personnel will obtain written informed consent by the participant. After study inclusion, each patient receives a structured systematic assessment by the study coordinator including (**Table 2**):

- Socio-demographic and anthropometric data (e.g. age, gender, weight and height, zip code [rural versus urban living area], marital status, nurse visit at home [German: Spitex], insurance coverage [private or not], admission mode [elective or urgent], transfer from intensive care unit [ICU], hospitalization in the last 6 months), previous use of mobility aids, comorbidities.

For peer review only - http://bmjopen.bmj.com/site/about/guidelines.xhtml

- Functional status using Barthel index. The Barthel index is one of the most widely used and reliable functional outcome measures and is measured through an established and validated questionnaire where higher scores on a scale between 0 and 100 indicate greater independence in activities of daily living.[31] It can be conducted by telephone and a validated German version is available.[32] If the patient is not able to answer the questions, the Barthel index will be administered by interviewing the ward nurse caring for the patient, a family member or any other person caring for the patient before hospitalization (e.g. Spitex).
- Concerns of falling using FES-I. FES-I is a validated 16-item tool to evaluate concerns of falling, with 16 score points indicating no concerns of falling and 64 score points indicating high levels of concern.[33] It can be conducted by telephone and a validated German version is available.[34]
- Quality of life will be assessed with the EQ-5D-5L questionnaire. The EQ-5D-5L[35 36] is an established, standardized measure of quality of life consisting of a descriptive system compromising the 5 dimensions mobility, self-care, usual activities, pain / discomfort, and anxiety / depression with five levels each (no problems, slight problems, moderate problems, severe problems, and extreme problems) and a visual analogue scale with 0 indicating the worst health and 100 indicating the best health.[39] It can be conducted by telephone interview and a validated German version is available.[37]
- Physical activity will be assessed by a trained study nurse using the DEMMI score. The German version of the DEMMI is a valid[40] and reliable[39] instrument to measure physical activity in older adults in the acute hospital setting and has no floor or ceiling effect.[29 38 41] Based on 15 items (0 to 19 points with Rasch conversion to a score ranging from 0 [indicating poor physical activity] to 100 [indicating a high level of independent physical activity]), the DEMMI includes evaluation of movement in bed (3 items), chair (3), static balance (4), walking (2), and dynamic balance (3). Acute geriatric patients with a DEMMI score below 41 score points had significantly lower

For peer review only - http://bmjopen.bmj.com/site/about/guidelines.xhtml

Barthel index (38% vs. 79%), used more mobility aids, were more bedridden, longer hospitalized, and were more often discharged to rehabilitation compared to those with a higher DEMMI score.[42] No association was found for gender, age, weight, body mass index, Charlson comorbidity index or handgrip strength.[42] Trained personnel is able to complete the DEMMI in no more than 10 minutes.

Table 2: Summary of study procedures based on Standard Protocol Items: Recommendations for Interventional Trials (SPIRIT) schedule. EHR, electronic health record.

	1st visit	2nd visit[1]	3rd visit	Phone call
**Time points** (days after randomization)	0	5	at discharge[2]	90
Oral and written patient information	+			
Written consent	+			
Inclusion-/exclusion criteria	+			
Physical activity (De Morton Mobility Index)	+	+	+	
Randomization	+			
Demographics	+			
Installation of accelerometer	+			
Read-out of accelerometer			+	
Number of in-hospital falls			+ / daily	
Number of delirium episodes (EHR)			+	
Length of stay (EHR)			+	
Medical history (EHR)			+	
Destination (EHR)			+	+
Concerns of falling (Falls Efficacy Scale – International, FES-I)	+			+
Independence during activities of daily living (Barthel-Index)	+			+
Quality of life (EQ-5D)	+			+
Number of falls (with / without injuries)				+
Number of re-hospitalisations				+
Mortality				+

[1] Omitted if 3rd visit before day 6
[2] latest on day 14

For peer review only - http://bmjopen.bmj.com/site/about/guidelines.xhtml

Mobility measurements

To objectively measure mobilization, patients will be equipped with a wrist-worn tri-axis accelerometer (GENEActiv, Activinsights, Kimbolton, Cambridgeshire, United Kingdom). This accelerometer has been validated and proven to reliably measure physical activity in hospitalized patients.[43 44] At inclusion, a study nurse will instruct the patients to wear the accelerometer as long as possible during hospitalization (day and night time) on either wrist side. The accelerometers will be collected before discharge or transfer to another hospital unit. The measurement frequency will be 50 Hz. Accelerometry data will be extracted and analyzed using the GGIR package for R.[45] Time mobilized (total minutes per day) will be calculated from time “moving” as total of time “inactive” and “static”, excluding time “not worn”. Mean acceleration in miliG/vector will be indicated.

Randomization and allocation procedures

After patient registration in the data entry system (REDCap) and confirmation of all inclusion and exclusion criteria, patients are randomized 1:1 to one of the two study groups. Randomization will be blocked using varying block sizes of 2, 4, and 6. Moreover, randomization will be stratified according to the baseline DEMMI score (≤ 40 vs > 40 points) and age (< 65 vs ≥ 65 years). The allocation sequence (randomization list) will be generated by an independent data manager at CTU Bern, University of Bern, Bern, Switzerland not otherwise involved in the trial, and implemented in the data entry system to ensure concealment of allocation.

Statistical analysis

The statistical analysis will be performed by CTU Bern, University of Bern, using the statistical software packages Stata or R. All recorded and derived variables will be presented using descriptive summary tables. Continuous variables will be summarized by mean and SD or median and interquartile range (IQR) as appropriate. Categorical variables will be summarized with absolute and relative frequencies.

For peer review only - http://bmjopen.bmj.com/site/about/guidelines.xhtml

Primary analysis will be intention-to-treat. The primary outcome change in DEMMI score from randomization to day 5 will be compared between both intervention groups using a linear model. The model will be adjusted for the baseline DEMMI score and stratification factors used for randomization.

Secondary continuous outcomes will be analyzed using the same approach as described above. To assess change in quality of life, we will analyse the difference between baseline and day 5 or day 14 in the visual analogue scale and the mobility dimension of the EQ-5D-5L. Count outcomes (number of delirium episodes, falls, and re-hospitalizations) will be assessed using a negative binomial model, categorical outcomes (destination) using a multinomial logistic model, binary outcomes (reaching MCID) using logistic regression, and time-to-event outcomes (LOS, mortality) using a Cox regression model. All models will be adjusted for stratification factors. In a per-protocol analysis, patients that violated any eligibility criteria, did not receive the allocated intervention, or were discharged before day 4 will be disregarded. All effect measures will be accompanied by 95% confidence intervals (CIs). The statistical testing will be two-sided with a type I error of 5%. The data analysts will be blinded to the group.

We will perform subgroup analysis for age groups <65 years vs. ≥65 years, initial performance in physical activity (DEMMI ≤40 vs. DEMMI >40), and pre-hospital mobility (no mobility aid vs. pre-hospital use of mobility aid) because previous studies showed that older age categories had significantly lower mean DEMMI scores[46], and lower Barthel index and use of mobility aids or being bedridden was associated with lower DEMMI scores.[42]

Any deviation from the original statistical plan will be described and justified in the final trial report. There is no interim analysis planned, i.e. there are no stopping rules on the individual or trial level.

Handling of missing data and drop-outs

If outcomes are missing, we will employ multiple imputation in the primary analysis and additionally perform an available case analysis as sensitivity analysis disregarding missing data. The DEMMI score that is measured two times during follow-up will additionally be

For peer review only - http://bmjopen.bmj.com/site/about/guidelines.xhtml

evaluated in a repeated measures mixed-effects linear model, additionally introducing a random intercept for patients into the model.

Interventions

Experimental group: In patients assigned to GDM, a trained physiotherapist will provide a short educational intervention on GDM. Further, the physiotherapist will define a personal mobility goal based on an adapted version of the Johns Hopkins Highest Level of Mobility (JH-HLM) scale (**Table 3, Supplementary Figure 1**).[11 20] To avoid a ceiling effect in our population, compared to the original version of the JH-HLM, we added the following mobility goal: “Level 8: Walking 75 m or more (30 min or stairs) and no bed rest during daytime” and skipped the original “Level 1: Only lying”.

Table 3: Comparison of Johns Hopkins Highest Level of Mobility Scale (JH-HLM) and GoMob-in mobility goal levels

Level	JH-HLM	GoMob-in mobility goal level
**1**	only lying	bed activities
**2**	bed activities	sit at edge of bed
**3**	sit at edge of bed	transfer to chair/commode
**4**	transfer to chair/commode	standing for >1 minute
**5**	standing for >1 minute	walking 10+ steps
**6**	walking 10+ steps	walking 7.5 m or more
**7**	walking 7.5 m or more	walking 75 m or more
**8**	walking 75 m or more	Walking 75 m or more (30 min or stairs) and no bed rest during daytime

Participants will be instructed, that they should aim for successful completion of the task at least 3 times daily. The mobility goal level will be defined regularly by the treating physiotherapist together with the patient taking into account the current physical activity. Re-assessment will serve as opportunity for a short motivational intervention (“booster session”).

For peer review only - http://bmjopen.bmj.com/site/about/guidelines.xhtml

The mobility goal level should be increased regularly. In patients reaching the highest mobility goal level, a note will be placed on the bed to indicate the patient to use the bed only for examinations and for sleep. All medical personnel (e.g., nurses) caring for the participant is allowed to adapt the mobility goal level. Increasing the mobility goal level more than one step or lowering it is also allowed. Nurses and physicians caring for participants in the intervention group will get informed regularly about the ongoing trial and its aims.

The mobility goal level will be depicted on a patient board next to the bed, visible for all stakeholders (patients, visiting friends and family, physicians, nurses, physiotherapists).

Control group: Patients assigned to the control group, will receive standard treatment of the GIM wards. Mobility goals will not be routinely set nor encouraged interprofessionally. Patients will receive physiotherapist's guided training as prescribed by the resident in charge. As in any exercise trial, physiotherapists providing the standard treatment can not be blinded to participation of the patients in the control group. They address the specific needs and provide general non-systematic information about the importance of mobilization during hospitalization as part of their routine instruction for all patients.

The adherence to intervention protocol will be assessed by presence of the patient board displaying the mobility goal level at discharge.

For peer review only

Follow-up

Nurses will record falls in the electronic health record and will be collected by the study nurse together with information on destination, LOS, diagnosis (delirium), number of medication and specific type (i.e., Anatomical Therapeutic Chemical [ATC] Classification System group) at the end of hospitalization.

Physical activity will be assessed using DEMMI in all participants on day 5 after study inclusion and on the day of discharge or day 14 (whichever occurs first).

Three months after study inclusion, all participants will be inquired by telephone interview for Barthel index, FES-I/, EQ-5D, number of falls since study inclusion (with / without injuries), current destination, re-hospitalizations and all-cause mortality. If the patient cannot be reached, a family member or the treating general practitioner will be called to complement

For peer review only - http://bmjopen.bmj.com/site/about/guidelines.xhtml

data. The patient will be asked to send the falls calendar to the study team. If discrepancy is noted compared to the phone interview, the participant will be called for validation.

Blinding

The DEMMI will be assessed by an independent physiotherapist not otherwise involved in the treatment of patients on the ward and to whom the group allocation of the participant is not declared. The patient will be instructed to not reveal the group allocation to the DEMMI assessor.

The statistician responsible for the final analysis will have no access to the group allocation until the primary analysis of the trial is finished. The data analysis will be conducted according to a pre-specified statistical analysis plan.

Data management

Study data will be collected and managed using REDCap electronic data capture tools hosted at CTU, University of Bern, Bern, Switzerland.[47 48] REDCap (Research Electronic Data Capture) is a secure, web-based software platform designed to support data capture for research studies, providing 1) an intuitive interface for validated data capture; 2) audit trails for tracking data manipulation and export procedures; 3) automated export procedures for seamless data downloads to common statistical packages; and 4) procedures for data integration and interoperability with external sources.

Safety

Protocol violations should not lead to treatment discontinuation unless they indicate a significant risk to patient safety. We will comply with all regulations concerning safety measures in clinical trials as set forth by the ethical committee. The investigators will report any serious adverse events occurring during clinical trials, independent of direct causal relationship with the intervention, within 24 hours.

Patient and public involvement

No patients were involved in the design of the study.

For peer review only - http://bmjopen.bmj.com/site/about/guidelines.xhtml

Ethics and dissemination

The trial will be performed in compliance with the Declaration of Helsinki and its amendments, and has been approved by the responsible Ethics Board (Ethikkommission Bern, Bern, Switzerland, 2020-02305). Important protocol modifications will be communicated to the Ethics Board and participating study sites. Potential participants must provide written informed consent before entering the study. Subjects can leave the study at any time for any reason without any negative consequences for their further treatment. When a patient ends the treatment phase of the study prematurely, we will record the date and reason for early treatment discontinuation. If possible, the end of treatment evaluations will be collected. Insurance coverage will be provided for all study participants by the study sponsor.

The results of the main trial and each of the secondary outcomes will be submitted for publication in open access peer-reviewed journals. The GoMob-in study group is an independent academic research group, which will not employ professional writers. Confidentiality of patients' data is ensured by all stakeholders at all times; patients will not be identified by names or identifiers such as birth date in any documents leaving the study site. Whenever data are exported from the database (e.g. for analysis), data will be coded.

Authors statement

FDL designed the study. MW, AL, MLV, JS, and JH critically commented on the methods and contributed to the development of the study. FDL wrote the first draft of the manuscript. All authors revised the manuscript and approved the final version.

Funding statement

The GoMob-in trial is funded by the Swiss Society of General Internal Medicine (SGAIM) Foundation, Bern, Switzerland (grant number: not applicable) and the Fondation Sana, Bern, Switzerland (grant number: GF 2021-0048).

Competing interests

The funder plays no role in the study design, collection of data, analysis, and writing of the manuscript. There is no conflict of interest regarding intellectual content and proprietary affairs.

For peer review only - http://bmjopen.bmj.com/site/about/guidelines.xhtml

The authors and investigators will have full access to the final trial dataset. No contractual agreements limit such access for investigators.

Data monitoring committee

Data monitoring will be conducted by the CTU Bern, University of Bern, Bern, Switzerland and include routine site monitoring visits according to a specific monitoring plan. Data collection, handling of the data, and the analysis will be done by the researchers at the University of Bern, Bern, Switzerland. The funder plays no role in the data acquisition, data storage, and analysis.

Data statement

Data will be available upon reasonable request.

Trial sponsor investigator

Fabian D. Liechti, MD, PhD

Department of General Internal Medicine

Bern University Hospital

Freiburgstrasse 8

3010 Bern

Mail: fabian.liechti@insel.ch

Phone: +41 31 632 21 11

For peer review only

References

1. Creditor MC. Hazards of hospitalization of the elderly. *Annals of internal medicine* 1993;118(3):219-23. doi: 10.7326/0003-4819-118-3-199302010-00011
2. Pashikanti L, Von Ah D. Impact of early mobilization protocol on the medical-surgical inpatient population: an integrated review of literature. *Clin Nurse Spec* 2012;26(2):87-94. doi: 10.1097/NUR.0b013e31824590e6 [published Online First: 2012/02/18]
3. Brown CJ, Friedkin RJ, Inouye SK. Prevalence and outcomes of low mobility in hospitalized older patients. *J Am Geriatr Soc* 2004;52(8):1263-70. doi: 10.1111/j.1532-5415.2004.52354.x [published Online First: 2004/07/24]
4. Zisberg A, Shadmi E, Sinoff G, et al. Low mobility during hospitalization and functional decline in older adults. *Journal of the American Geriatrics Society* 2011;59(2):266-73. doi: 10.1111/j.1532-5415.2010.03276.x
5. Kortebein P, Symons TB, Ferrando A, et al. Functional impact of 10 days of bed rest in healthy older adults. *The journals of gerontology Series A, Biological sciences and medical sciences* 2008;63(10):1076-81. doi: 10.1093/gerona/63.10.1076
6. Growdon ME, Shorr RI, Inouye SK. The Tension Between Promoting Mobility and Preventing Falls in the Hospital. *JAMA internal medicine* 2017;177(6):759-60. doi: 10.1001/jamainternmed.2017.0840
7. Brown CJ, Foley KT, Lowman JD, Jr., et al. Comparison of Posthospitalization Function and Community Mobility in Hospital Mobility Program and Usual Care Patients: A Randomized Clinical Trial. *JAMA internal medicine* 2016;176(7):921-7. doi: 10.1001/jamainternmed.2016.1870
8. Cohen Y, Zisberg A, Chayat Y, et al. Walking for Better Outcomes and Recovery: The Effect of WALK-FOR in Preventing Hospital-Associated Functional Decline

For peer review only

Among Older Adults. *The journals of gerontology Series A, Biological sciences and medical sciences* 2019;74(10):1664-70. doi: 10.1093/gerona/glz025

9. Hunter A, Johnson L, Coustasse A. Reduction of intensive care unit length of stay: the case of early mobilization. *The health care manager* 2014;33(2):128-35. doi: 10.1097/HCM.0000000000000006

10. Moore JE, Liu B, Khan S, et al. Can the effects of the mobilization of vulnerable elders in Ontario (MOVE ON) implementation be replicated in new settings: an interrupted time series design. *BMC geriatrics* 2019;19(1):99. doi: 10.1186/s12877-019-1124-0

11. Hoyer EH, Young DL, Klein LM, et al. Toward a Common Language for Measuring Patient Mobility in the Hospital: Reliability and Construct Validity of Interprofessional Mobility Measures. *Physical therapy* 2018;98(2):133-42. doi: 10.1093/ptj/pzx110

12. Smart DA, Dermody G, Coronado ME, et al. Mobility Programs for the Hospitalized Older Adult: A Scoping Review. *Gerontology & geriatric medicine* 2018;4:2333721418808146. doi: 10.1177/2333721418808146

13. Mudge AM, McRae P, McHugh K, et al. Poor mobility in hospitalized adults of all ages. *J Hosp Med* 2016;11(4):289-91. doi: 10.1002/jhm.2536 [published Online First: 2016/01/23]

14. Brown CJ, Redden DT, Flood KL, et al. The underrecognized epidemic of low mobility during hospitalization of older adults. *J Am Geriatr Soc* 2009;57(9):1660-5. doi: 10.1111/j.1532-5415.2009.02393.x [published Online First: 2009/08/18]

15. Valkenet K, Bor P, van Delft L, et al. Measuring physical activity levels in hospitalized patients: a comparison between behavioural mapping and data

For peer review only

For peer review only - http://bmjopen.bmj.com/site/about/guidelines.xhtml

from an accelerometer. *Clinical rehabilitation* 2019;33(7):1233-40. doi: 10.1177/0269215519836454

16. Liu B, Moore JE, Almaawiy U, et al. Outcomes of Mobilisation of Vulnerable Elders in Ontario (MOVE ON): a multisite interrupted time series evaluation of an implementation intervention to increase patient mobilisation. *Age and ageing* 2018;47(1):112-19. doi: 10.1093/ageing/afx128

17. Hashem MD, Parker AM, Needham DM. Early Mobilization and Rehabilitation of Patients Who Are Critically Ill. *Chest* 2016;150(3):722-31. doi: 10.1016/j.chest.2016.03.003

18. Cameron S, Ball I, Cepinskas G, et al. Early mobilization in the critical care unit: A review of adult and pediatric literature. *Journal of critical care* 2015;30(4):664-72. doi: 10.1016/j.jcrc.2015.03.032

19. Hurn J, Kneebone I, Cropley M. Goal setting as an outcome measure: A systematic review. *Clin Rehabil* 2006;20(9):756-72. doi: 10.1177/0269215506070793 [published Online First: 2006/09/29]

20. Hoyer EH, Friedman M, Lavezza A, et al. Promoting mobility and reducing length of stay in hospitalized general medicine patients: A quality-improvement project. *J Hosp Med* 2016;11(5):341-7. doi: 10.1002/jhm.2546 [published Online First: 2016/02/06]

21. Exum E, Hull BL. The Process of Implementing a Mobility Technician in the General Medicine and Surgical Population to Increase Patient Mobility and Improve Hospital Quality Measures: A Pilot Study. *Journal of Acute Care Physical Therapy* 2019;10(4) doi: 10.1097/JAT.0000000000000110

22. Klein LM, Young D, Feng D, et al. Increasing patient mobility through an individualized goal-centered hospital mobility program: A quasi-experimental

For peer review only - http://bmjopen.bmj.com/site/about/guidelines.xhtml

quality improvement project. *Nurs Outlook* 2018;66(3):254-62. doi: 10.1016/j.outlook.2018.02.006 [published Online First: 2018/05/01]

23. Teodoro CR, Breault K, Garvey C, et al. STEP-UP: Study of the Effectiveness of a Patient Ambulation Protocol. *Medsurg Nurs* 2016;25(2):111-6. [published Online First: 2016/06/22]

24. Cortes OL, Delgado S, Esparza M. Systematic review and meta-analysis of experimental studies: In-hospital mobilization for patients admitted for medical treatment. *J Adv Nurs* 2019;75(9):1823-37. doi: 10.1111/jan.13958 [published Online First: 2019/01/24]

25. Chan AW, Tetzlaff JM, Gøtzsche PC, et al. SPIRIT 2013 explanation and elaboration: guidance for protocols of clinical trials. *BMJ (Clinical research ed)* 2013;346:e7586. doi: 10.1136/bmj.e7586 [published Online First: 2013/01/11]

26. Reisberg B, Ferris SH, de Leon MJ, et al. The Global Deterioration Scale for assessment of primary degenerative dementia. *Am J Psychiatry* 1982;139(9):1136-9. doi: 10.1176/ajp.139.9.1136

27. Karlsen A, Loeb MR, Andersen KB, et al. Improved Functional Performance in Geriatric Patients during Hospital Stay. *American Journal of Physical Medicine and Rehabilitation* 2017;96(5):e78-e84. doi: 10.1097/PHM.0000000000000671

28. Oestergaard AS, Mathiesen MH, Karlsen A, et al. In acutely admitted geriatric patients, offering increased physical activity during hospitalization decreases length of stay and can improve mobility. *Translational Sports Medicine* 2018;1(1):46-53. doi: 10.1002/tsm2.2

29. de Morton NA, Davidson M, Keating JL. The de Morton Mobility Index (DEMMI): an essential health index for an ageing world. *Health and quality of life outcomes* 2008;6:63. doi: 10.1186/1477-7525-6-63

For peer review only - http://bmjopen.bmj.com/site/about/guidelines.xhtml

30. Pedersen MM, Bodilsen AC, Petersen J, et al. Twenty-four-hour mobility during acute hospitalization in older medical patients. *J Gerontol A Biol Sci Med Sci* 2013;68(3):331-7. doi: 10.1093/gerona/gls165 [published Online First: 2012/09/14]

31. Richards SH, Peters TJ, Coast J, et al. Inter-rater reliability of the Barthel ADL index: how does a researcher compare to a nurse? *Clinical rehabilitation* 2000;14(1):72-8. doi: 10.1191/026921500667059345

32. Lubke N, Meinck M, Von Renteln-Kruse W. [The Barthel Index in geriatrics. A context analysis for the Hamburg Classification Manual]. *Zeitschrift fur Gerontologie und Geriatrie* 2004;37(4):316-26. doi: 10.1007/s00391-004-0233-2

33. Yardley L, Beyer N, Hauer K, et al. Development and initial validation of the Falls Efficacy Scale-International (FES-I). *Age and ageing* 2005;34(6):614-9. doi: 10.1093/ageing/afi196

34. Dias N, Kempen GI, Todd CJ, et al. [The German version of the Falls Efficacy Scale-International Version (FES-I)]. *Zeitschrift fur Gerontologie und Geriatrie* 2006;39(4):297-300. doi: 10.1007/s00391-006-0400-8

35. EuroQol G. EuroQol--a new facility for the measurement of health-related quality of life. *Health policy* 1990;16(3):199-208. doi: 10.1016/0168-8510(90)90421-9

36. Foundation ER. EQ-5D-5L User Guide, Version 3.0 2019 [updated 09/2019. Available from: https://euroqol.org/publications/user-guides accessed 08.07.2020.

37. Ludwig K, Graf von der Schulenburg JM, Greiner W. German Value Set for the EQ-5D-5L. *PharmacoEconomics* 2018;36(6):663-74. doi: 10.1007/s40273-018-0615-8

38. de Morton NA, Davidson M, Keating JL. Validity, responsiveness and the minimal clinically important difference for the de Morton Mobility Index (DEMMI) in an

For peer review only - http://bmjopen.bmj.com/site/about/guidelines.xhtml

older acute medical population. *BMC Geriatr* 2010;10:72. doi: 10.1186/1471-2318-10-72 [published Online First: 2010/10/06]

39. Dasenbrock L, Berg T, Lurz S, et al. [The De Morton Mobility Index for evaluation of early geriatric rehabilitation]. *Z Gerontol Geriatr* 2016;49(5):398-404. doi: 10.1007/s00391-016-1061-x

40. Braun T, Schulz RJ, Reinke J, et al. Reliability and validity of the German translation of the de Morton Mobility Index (DEMMI) performed by physiotherapists in patients admitted to a sub-acute inpatient geriatric rehabilitation hospital. *BMC geriatrics* 2015;15:58. doi: 10.1186/s12877-015-0035-y

41. de Morton NA, Davidson M, Keating JL. Reliability of the de Morton Mobility Index (DEMMI) in an older acute medical population. *Physiotherapy research international : the journal for researchers and clinicians in physical therapy* 2011;16(3):159-69. doi: 10.1002/pri.493

42. Melgaard D, Rodrigo-Domingo M, Morch MM, et al. DEMMI Scores, Length of Stay, and 30-Day Readmission of Acute Geriatric Patients in Denmark: A Cross-Sectional Observational Study with Longitudinal Follow-Up. *Geriatrics* 2019;4(1) doi: 10.3390/geriatrics4010008

43. Esliger DW, Rowlands AV, Hurst TL, et al. Validation of the GENEA Accelerometer. *Med Sci Sports Exerc* 2011;43(6):1085-93. doi: 10.1249/MSS.0b013e31820513be

44. Rowlands AV, Olds TS, Hillsdon M, et al. Assessing sedentary behavior with the GENEActiv: introducing the sedentary sphere. *Med Sci Sports Exerc* 2014;46(6):1235-47. doi: 10.1249/MSS.0000000000000224

For peer review only

For peer review only - http://bmjopen.bmj.com/site/about/guidelines.xhtml

45. Rowlands AV, Yates T, Davies M, et al. Raw Accelerometer Data Analysis with GGIR R-package: Does Accelerometer Brand Matter? *Med Sci Sports Exerc* 2016;48(10):1935-41. doi: 10.1249/MSS.0000000000000978

46. Macri EM, Lewis JA, Khan KM, et al. The de morton mobility index: normative data for a clinically useful mobility instrument. *Journal of aging research* 2012;2012:353252. doi: 10.1155/2012/353252

47. Harris PA, Taylor R, Thielke R, et al. Research electronic data capture (REDCap)--a metadata-driven methodology and workflow process for providing translational research informatics support. *J Biomed Inform* 2009;42(2):377-81. doi: 10.1016/j.jbi.2008.08.010 [published Online First: 2008/10/22]

48. Harris PA, Taylor R, Minor BL, et al. The REDCap consortium: Building an international community of software platform partners. *J Biomed Inform* 2019;95:103208. doi: 10.1016/j.jbi.2019.103208 [published Online First: 2019/05/13]

For peer review only

For peer review only - http://bmjopen.bmj.com/site/about/guidelines.xhtml

Figure Title and Legend

Figure 1. Study flow chart. DEMMI, de Morton Mobility Index; EHR, electronic health record; R, randomisation.

Supplementary Figure 1. GoMob-in mobility goal levels as attached to the patient's board close to its bed and icons used to indicate mobility restrictions.

For peer review only

For peer review only - http://bmjopen.bmj.com/site/about/guidelines.xhtml

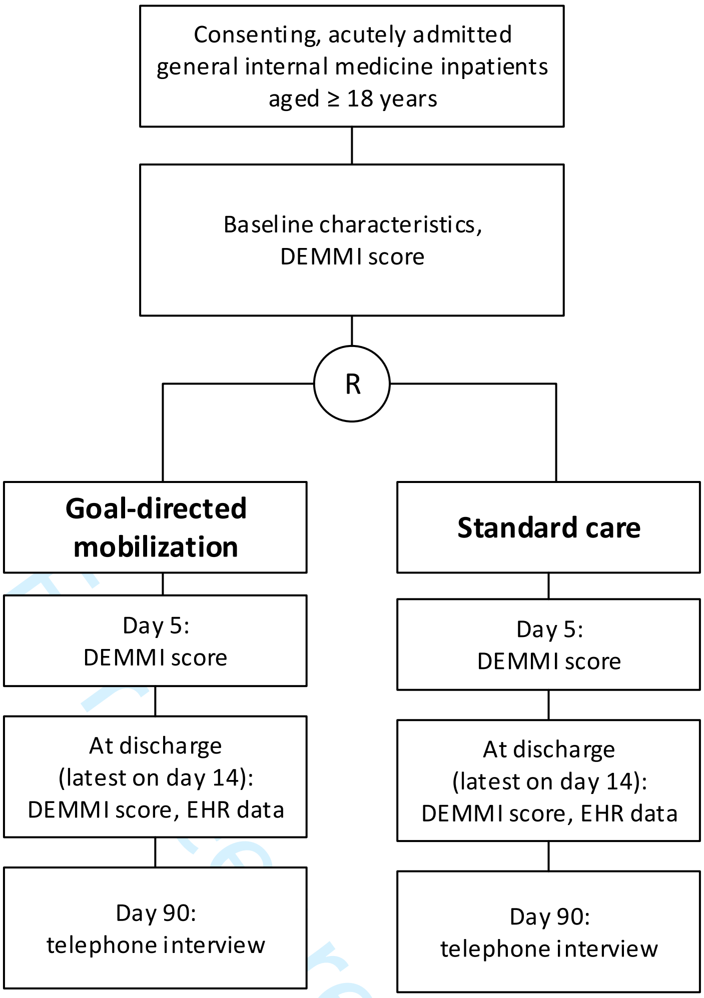

For peer review only

Stufe	Tages-Ziel (mindestens 3 x/Tag)
8	≥ 75 m gehen (30 min oder Treppe) & keine Bettruhe
7	≥ 75 m gehen
6	≥ 7,5 m gehen
5	≥ 10 Schritte gehen
4	≥ 1 Minute stehen
3	Transfer auf den Stuhl / Nachtstuhl
2	An die Bettkante sitzen
1	Aktivitäten im Bett / unselbständiger Transfer

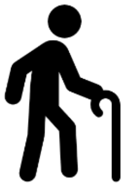
mit Gehstock

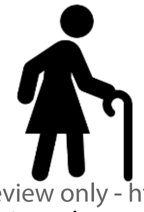
mit Gehstock

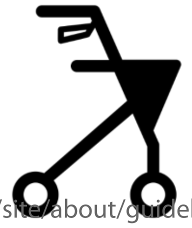
mit Rollator

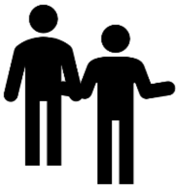
in Begleitung

For peer review only - http://bmjopen.bmj.com/site/about/guidelines.xhtml

Klinik für Allgemeine Innere Medizin
Inselspital, 3010 Bern

Ziel-gerichtete Mobilisierung von Spitalpatienten (Goal-directed Mobilization of Medical inpatients – GoMob-in)

Diese Studie ist organisiert durch: Dr. med. Fabian D. Liechti, MD-PhD
Klinik für Allgemeine Innere Medizin KAIM
Inselspital / Universität Bern
Freiburgstrasse, 3010 Bern

Sehr geehrte Dame, sehr geehrter Herr,

Wir möchten Sie anfragen, ob Sie an einer klinischen Studie teilnehmen wollen. Im Folgenden wird Ihnen dieses Studienvorhaben dargestellt.

Detailliertere Information

1. Ziel der Studie

Wir möchten Sie hiermit bitten, an unserer klinischen Studie teilzunehmen. Die Studie untersucht, wie die Mobilität von Patienten im Spital verbessert werden könnte. Wir machen diese Studie, um herauszufinden, ob eine tägliche Zielvorgabe bei hospitalisierten Patienten Beweglichkeit, Lebensqualität und Überleben verbessert und Stürze, Verwirrtheit und Wiedereintritte ins Spital reduziert.

2. Auswahl

Es können alle Personen teilnehmen, die auf der Bettenstation der Allgemeinen Inneren Medizin des Inselspitals hospitalisiert werden (Eintritt vor weniger als zwei Tagen) und Physiotherapie durch die Stationsärztin oder den Stationsarzt verordnet erhalten haben. Ausserdem müssen Sie diese Einverständniserklärung verstehen und unterschreiben können. Nicht teilnehmen dürfen Personen welche voraussichtlich weniger als 5 Tage hospitalisiert werden, eine relevante Geheinschränkung (wie z. B. einen Beinbruch oder einen Schlaganfall mit Halbseitenlähmung) haben, vom Arzt Bettruhe verordnet bekommen oder schwanger/stillend sind.

3. Allgemeine Informationen

Viele Patienten, welche auf einer Bettenstation hospitalisiert werden, bewegen sich während des Spitalaufenthaltes wenig. Es ist bekannt, dass dieser Bewegungsmangel zu Komplikationen wie Muskelabbau, Stürzen, Verwirrtheit und verschlechterter Bewegungsfähigkeit führen kann. Deswegen wird empfohlen, dass sich die Patienten während der Hospitalisation viel bewegen – abhängig vom Gesundheitszustand und allenfalls durch den behandelnden Arzt verfügter Einschränkung.

Diese Studie wird an der Klinik für Allgemeine Innere Medizin am Inselspital Bern durchgeführt. Wir planen 160 Personen in die Studie einzuschliessen. In dieser Studie wollen wir klären, ob eine klare Kommunikation und Angabe eines individuellen Tagesziels die Beweglichkeit von hospitalisierten Personen verbessern kann.

Die Teilnehmenden werden zufällig einer von 2 Gruppen zugeordnet: einer Motivations-Gruppe und einer Kontroll-Gruppe. Die Wahrscheinlichkeit der Zuteilung auf eine Gruppe beträgt 50%. Zur Messung der Bewegung, wird Ihnen während des Spitalaufenthaltes ein Schrittzähler angehängt. Dieser misst Ihre Bewegung, woraus ein Computerprogramm berechnen kann, ob sie schlafen, stehen, sitzen oder gehen. In der Motivations-Gruppe wird den Patienten jeden Tag vorgegeben,

For peer review only - http://bmjopen.bmj.com/site/about/guidelines.xhtml

wie viel sie sich mindestens bewegen sollen. In der Kontroll-Gruppe bewegen sich die Patienten unabhängig von einem Tagesziel.

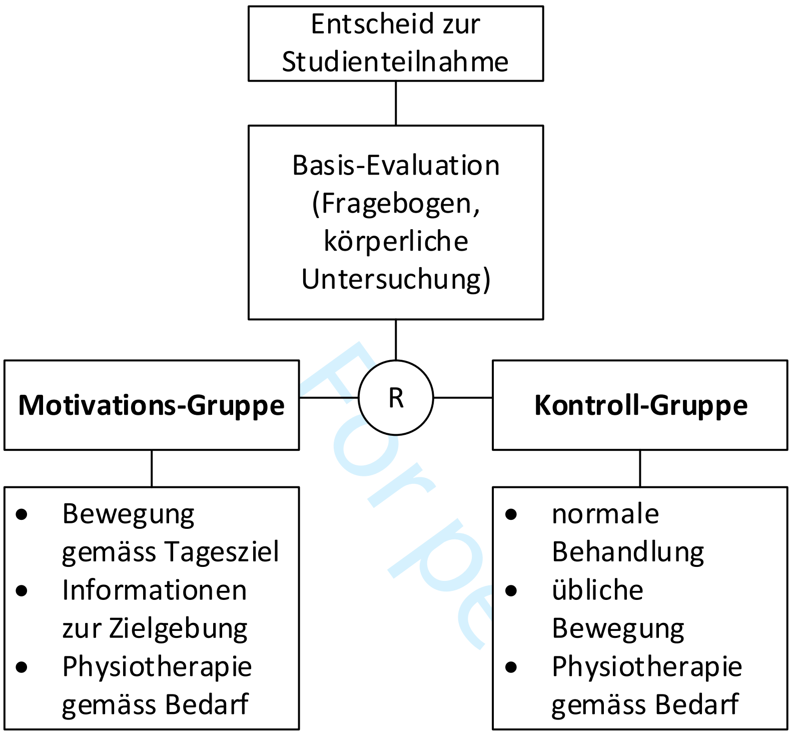

Abbildung 1: Übersicht der beiden Gruppen. (R = zufällige Zuordnung zu einer der beiden Gruppen)

Die aktive Teilnahme dauert solange wie der Spitalaufenthalt, jedoch höchstens 14 Tage und wird mit einem Fragebogen welcher nach 3 Monaten telefonisch beantwortet wird, abgeschlossen.

Wir machen diese Studie so, wie es die Gesetze in der Schweiz vorschreiben. Ausserdem beachten wir alle international anerkannten Richtlinien. Die zuständige Kantonale Ethikkommission hat die Studie geprüft und bewilligt.

Eine Beschreibung dieser Studie finden Sie auch auf der Internetseite des Bundesamtes für Gesundheit: www.kofam.ch

4. Ablauf

Nach Einwilligung zur Teilnahme an der Studie wird eine Eintrittsbefragung und -Untersuchung durchgeführt. Diese beinhaltet folgendes:

- Überprüfung der Ein- und Ausschlusskriterien der Studie
- Fragen zu Ihrer Person wie Grösse, Gewicht, Wohnort, Gehhilfen, Vorerkrankungen, etc.
- Fragebogen zur Gangunsicherheit, Selbständigkeit und Lebensqualität (ca. 30 min)
- Körperliche Untersuchung Ihrer Beweglichkeit (ca. 10-15 min)

Für die Studie sind keine zusätzlichen Blutentnahmen oder Röntgenuntersuchungen notwendig. Bei Frauen im gebährfähigen Alter wird aus einer bereits vorhandenen Blutprobe das Schwangerschaftshormon analysiert um eine Schwangerschaft auszuschliessen.

Die Untersuchung der Beweglichkeit wird am fünften Tag und vor Spitalaustritt oder spätestens nach 14 Tagen wiederholt. Sie müssen also für die Studie nicht länger im Spital bleiben als sonst vorgesehen.
Angaben zu ihrer Gesundheit wie z. B. Diagnosen, eingenommene Medikamente, Stürze werden wir zudem nach Ihrem Austritt aus Ihrer elektronischen Krankenakte entnehmen.

For peer review only - http://bmjopen.bmj.com/site/about/guidelines.xhtml

Klinik für Allgemeine Innere Medizin
Inselspital, 3010 Bern

Wir werden ihnen ein Sturztagebuch mit nach Hause geben, wo sie allfällige Stürze dokumentieren können. Nach 3 Monaten werden wir Sie telefonisch kontaktieren um einige Fragen zu Ihrer Gesundheit zu stellen (Fragebogen zur Lebensqualität, zur Gangunsicherheit, zur Selbständigkeit, zum aktuellen Aufenthaltsort u. ä.). Sollten wir Sie wiederholt nicht erreichen, werden wir Ihre Angehörigen oder Ihren Hausarzt kontaktieren, um diese Fragen klären zu können.

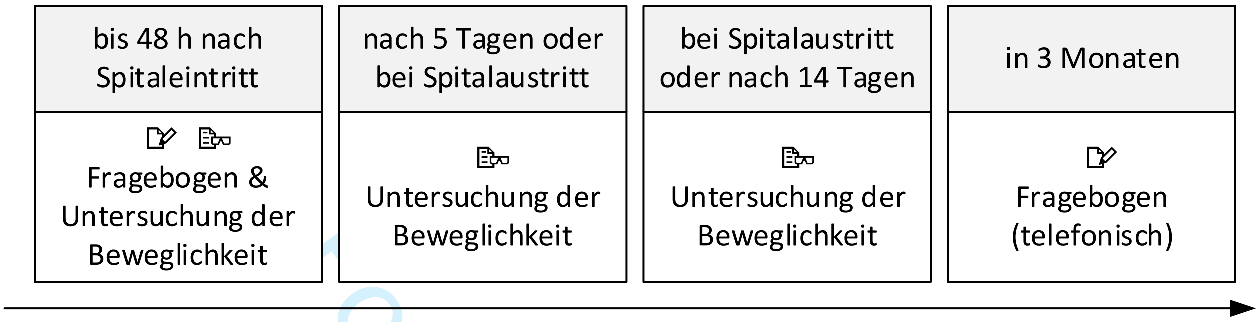

Abbildung 2: Ablauf der Untersuchungen während der Studie

Es kann sein, dass wir Sie von der Studie vorzeitig ausschliessen müssen. Das kann geschehen, wenn die Studie vorzeitig abgebrochen wird oder wir bei Ihnen ein Ausschlusskriterium finden, welches bei Einschluss nicht bekannt war.

5. Nutzen

Die Resultate der Studie können wichtig sein zur Erforschung der Mobilität bei Spitalpatienten.

6. Rechte

Sie nehmen freiwillig teil. Wenn Sie nicht mitmachen oder später Ihre Teilnahme zurückziehen wollen, müssen Sie dies nicht begründen. Ihre medizinische Behandlung/Betreuung ist unabhängig von Ihrem Entscheid gewährleistet. Sie dürfen jederzeit Fragen zur Studienteilnahme stellen. Wenden Sie sich dazu bitte an die Person, die am Ende dieser Information genannt ist.

7. Pflichten

Als Teilnehmer ist es notwendig, dass Sie
- sich an die notwendigen Vorgaben und Anforderungen der Studie durch den Prüfplan halten,
- Ihren Prüfarzt über den Verlauf der Erkrankung informieren und neue Symptome, neue Beschwerden und Änderungen im Befinden melden.

8. Risiken und Belastungen für die Teilnehmenden

Es sind keine Risiken der Studienintervention zu erwarten.

9. Andere Behandlungsmöglichkeiten

Sie müssen bei dieser Studie nicht teilnehmen. Wenn Sie nicht mitmachen, können Sie z. B. selbst auf eine ausreichende Bewegung während des Spitalaufenthalts achten.

10. Ergebnisse aus der Studie

Der Prüfarzt wird Sie während der Studie über alle neuen Erkenntnisse informieren, die den Nutzen der Studie oder Ihre Sicherheit und somit Ihr Einwilligung zur Teilnahme an der Studie beeinflussen können. Sie werden die Information mündlich und schriftlich erhalten.

11. Vertraulichkeit der Daten

Für diese Studie werden Ihre persönlichen und medizinischen Daten erfasst. Nur sehr wenige Fachpersonen werden Ihre unverschlüsselten Daten sehen, und zwar ausschliesslich, um Aufgaben im Rahmen der Studie zu erfüllen. Bei der Datenerhebung zu Studienzwecken werden die Daten verschlüsselt. Verschlüsselung bedeutet, dass alle Bezugsdaten, die Sie identifizieren

For peer review only - http://bmjopen.bmj.com/site/about/guidelines.xhtml

könnten (Name, Geburtsdatum), gelöscht und durch einen Schlüssel ersetzt werden. Die Schlüssel-Liste bleibt immer im Inselspital. Diejenigen Personen, die den Schlüssel nicht kennen, können daher keine Rückschlüsse auf Ihre Person ziehen. Bei einer Publikation sind die zusammengefassten Daten daher auch nicht auf Sie als Einzelperson rückverfolgbar. Ihr Name taucht niemals im Internet oder einer Publikation auf. Manchmal gibt es die Vorgabe zur Publikation bei einer Zeitschrift, dass Einzel-Daten (sogenannte Roh-Daten) übermittelt werden müssen. Wenn Einzel-Daten übermittelt werden müssen, dann sind die Daten immer verschlüsselt und somit ebenfalls nicht zu Ihnen als Person rückverfolgbar. Alle Personen, die im Rahmen der Studie Einsicht in Ihre Daten haben, unterliegen der Schweigepflicht. Die Vorgaben des Datenschutzes werden eingehalten und Sie als teilnehmende Person haben jederzeit das Recht auf Einsicht in Ihre Daten.
Möglicherweise wird diese Studie durch die zuständige Ethikkommission überprüft. Der Prüfarzt muss eventuell Ihre persönlichen und medizinischen Daten für solche Kontrollen offenlegen. Alle Personen müssen absolute Vertraulichkeit wahren.
Es ist möglich, dass Ihr nachbehandelnder Arzt kontaktiert wird, um Auskunft über Ihren Gesundheitszustand zu geben.

12. Rücktritt
Sie können jederzeit aufhören und von der Studie zurücktreten, wenn Sie das wünschen. Die bis dahin erhobenen Daten werden noch verschlüsselt ausgewertet, weil das ganze Projekt sonst seinen Wert verliert.
Nach der Auswertung werden Ihre Daten vollständig anonymisiert, d.h. Ihre Schlüsselzuordnung wird vernichtet, so dass danach niemand mehr erfahren kann, dass die Daten ursprünglich von Ihnen stammten.

13. Entschädigung für Teilnehmende
Wenn Sie an dieser Studie teilnehmen, bekommen Sie dafür keine Entschädigung. Es entstehen Ihnen oder Ihrer Krankenkasse keine Kosten durch die Teilnahme.

14. Haftung
Die Institution, die die Studie veranlasst hat und für die Durchführung verantwortlich ist, haftet für Schäden, welche Ihnen im Zusammenhang mit den Forschungshandlungen (z.B. Untersuchungen) entstehen könnten. Die Voraussetzungen und das Vorgehen dazu sind gesetzlich geregelt.
Falls Sie einen Schaden erlitten haben, so wenden Sie sich bitte an den Prüfarzt.

15. Finanzierung der Studie
Die Studie wird grösstenteils von der SGAIM Foundation (Stiftung der Schweizerischen Gesellschaft für Allgemeine Innere Medizin) bezahlt.

16. Kontaktperson(en)
Bei Fragen, Unsicherheiten oder Notfällen, die während der Studie oder danach auftreten, können Sie sich jederzeit an eine dieser Kontaktpersonen wenden.

Prüfarzt: Dr. med. Fabian D. Liechti, MD-PhD
Klinik für Allgemeine Innere Medizin KAIM
Inselspital / Universität Bern
Freiburgstrasse, 3010 Bern
fabian.liechti@insel.ch
Tel. +41 31 632 21 11 / Sucher: 181-7253

Klinik für Allgemeine Innere Medizin
Inselspital, 3010 Bern

Einwilligungserklärung

Schriftliche Einwilligungserklärung zur Teilnahme an einem Studienprojekt
Bitte lesen Sie dieses Formular sorgfältig durch. Bitte fragen Sie, wenn Sie etwas nicht verstehen oder wissen möchten. Für die Teilnahme ist Ihre schriftliche Einwilligung notwendig.

**BASEC-Nummer (nach Einreichung):**	
**Titel der Studie (wissenschaftlich und Laiensprache)**:	Ziel-gerichtete Mobilisierung von Spitalpatienten (Goal-directed Mobilization of Medical inpatients – GoMob-in)
**verantwortliche Institution (Sponsor mit Adresse)**:	Inselspital, Klinik für Allgemeine Innere Medizin, Freiburgstrasse, 3010 Bern
**Ort der Durchführung**:	Bern
**Verantwortlicher Prüfarzt / Studienkoordinator am Studienort:** Name und Vorname in Druckbuchstaben:	Dr. med. Fabian D. Liechti
**Teilnehmerin/Teilnehmer:** Name und Vorname in Druckbuchstaben: Geburtsdatum:	☐ weiblich ☐ männlich

- Ich wurde vom unterzeichnenden Prüfarzt mündlich und schriftlich über den Zweck, den Ablauf der Studie, über mögliche Vor- und Nachteile sowie über eventuelle Risiken informiert.
- Ich nehme an dieser Studie freiwillig teil und akzeptiere den Inhalt der abgegebenen schriftlichen Information. Ich hatte genügend Zeit, meine Entscheidung zu treffen.
- Ich wurde über mögliche andere Behandlungen und Behandlungsverfahren aufgeklärt.
- Meine Fragen im Zusammenhang mit der Teilnahme an dieser Studie sind mir beantwortet worden. Ich behalte die schriftliche Information und erhalte eine Kopie meiner schriftlichen Einwilligungserklärung.
- Ich bin mir bewusst, dass die in der Teilnehmerinformation genannten Pflichten einzuhalten sind. Im Interesse meiner Gesundheit kann mich der Prüfarzt jederzeit von der Studie ausschliessen.
- Ich bin einverstanden, dass mein Hausarzt über meine Teilnahme an der Studie informiert wird.
- Ich bin einverstanden, dass die zuständigen Fachleute des Sponsors, der zuständigen Ethikkommission zu Prüf- und Kontrollzwecken in meine unverschlüsselten Daten Einsicht nehmen dürfen, jedoch unter strikter Einhaltung der Vertraulichkeit.
- Bei Studienergebnissen, die direkt meine Gesundheit betreffen, werde ich informiert. Wenn ich das nicht wünsche, informiere ich meinen Prüfarzt.
- Ich weiss, dass meine gesundheitsbezogenen und persönlichen Daten nur in verschlüsselter Form zu Forschungszwecken für diese Studie weitergegeben werden können.
- Ich kann jederzeit und ohne Angabe von Gründen von der Studienteilnahme zurücktreten. Meine weitere medizinische Behandlung ist unabhängig von der Studienteilnahme immer gewährleistet. Die bis zum Rücktritt erhobenen Daten werden für die Auswertung zur Studie verwendet.
- Die Haftpflichtversicherung des Spitals/der Institution kommt für allfällige Schäden auf.

For peer review only

Klinik für Allgemeine Innere Medizin
Inselspital, 3010 Bern

Ort, Datum	Unterschrift Teilnehmerin/Teilnehmer

Bestätigung des Prüfarztes/der Prüfperson: Hiermit bestätige ich, dass ich dieser Teilnehmerin/ diesem Teilnehmer Wesen, Bedeutung und Tragweite der Studie erläutert habe. Ich versichere, alle im Zusammenhang mit dieser Studie stehenden Verpflichtungen gemäss dem geltenden Recht zu erfüllen. Sollte ich zu irgendeinem Zeitpunkt während der Durchführung der Studie von Aspekten erfahren, welche die Bereitschaft der Teilnehmerin/ des Teilnehmers zur Teilnahme an der Studie beeinflussen könnten, werde ich sie/ ihn umgehend darüber informieren.

Ort, Datum	Name und Vorname der Prüfärztin / des Prüfarztes / des Studienkoordinators / der Studienkoordinatorin in Druckbuchstaben<br><br>Unterschrift der Prüfärztin/des Prüfarztes des Studienkoordinators / der Studienkoordinatorin

For peer review only

SPIRIT

STANDARD PROTOCOL ITEMS: RECOMMENDATIONS FOR INTERVENTIONAL TRIALS

SPIRIT 2013 Checklist: Recommended items to address in a clinical trial protocol and related documents*

Section/item	Item No	Description
**Administrative information**		
Title	1	Descriptive title identifying the study design, population, interventions, and, if applicable, trial acronym **(p. 1)**
Trial registration	2a	Trial identifier and registry name. If not yet registered, name of intended registry **(p. 5)**
	2b	All items from the World Health Organization Trial Registration Data Set **(p. 5-7)**
Protocol version	3	Date and version identifier **(p. 3)**
Funding	4	Sources and types of financial, material, and other support **(p. 19)**
Roles and responsibilities	5a	Names, affiliations, and roles of protocol contributors **(p. 19)**
	5b	Name and contact information for the trial sponsor **(p. 19)**
	5c	Role of study sponsor and funders, if any, in study design; collection, management, analysis, and interpretation of data; writing of the report; and the decision to submit the report for publication, including whether they will have ultimate authority over any of these activities **(p. 19)**
	5d	Composition, roles, and responsibilities of the coordinating centre, steering committee, endpoint adjudication committee, data management team, and other individuals or groups overseeing the trial, if applicable (see Item 21a for data monitoring committee) **(p. 19)**
**Introduction**		
Background and rationale	6a	Description of research question and justification for undertaking the trial, including summary of relevant studies (published and unpublished) examining benefits and harms for each intervention **(p. 4)**
	6b	Explanation for choice of comparators **(p. 4-5)**
Objectives	7	Specific objectives or hypotheses **(p. 7)**

For peer review only

Trial design	8	Description of trial design including type of trial (eg, parallel group, crossover, factorial, single group), allocation ratio, and framework (eg, superiority, equivalence, noninferiority, exploratory) **(p. 7, 13)**

Methods: Participants, interventions, and outcomes

Study setting	9	Description of study settings (eg, community clinic, academic hospital) and list of countries where data will be collected. Reference to where list of study sites can be obtained **(p. 7)**
Eligibility criteria	10	Inclusion and exclusion criteria for participants. If applicable, eligibility criteria for study centres and individuals who will perform the interventions (eg, surgeons, psychotherapists) **(p. 7-8)**
Interventions	11a	Interventions for each group with sufficient detail to allow replication, including how and when they will be administered **(p. 14-16)**
	11b	Criteria for discontinuing or modifying allocated interventions for a given trial participant (eg, drug dose change in response to harms, participant request, or improving/worsening disease) **(p. 17)**
	11c	Strategies to improve adherence to intervention protocols, and any procedures for monitoring adherence (eg, drug tablet return, laboratory tests) **(p. 16)**
	11d	Relevant concomitant care and interventions that are permitted or prohibited during the trial **(p. 16)**
Outcomes	12	Primary, secondary, and other outcomes, including the specific measurement variable (eg, systolic blood pressure), analysis metric (eg, change from baseline, final value, time to event), method of aggregation (eg, median, proportion), and time point for each outcome. Explanation of the clinical relevance of chosen efficacy and harm outcomes is strongly recommended **(p. 8-9)**
Participant timeline	13	Time schedule of enrolment, interventions (including any run-ins and washouts), assessments, and visits for participants. A schematic diagram is highly recommended (see Figure) **(p. 12)**
Sample size	14	Estimated number of participants needed to achieve study objectives and how it was determined, including clinical and statistical assumptions supporting any sample size calculations **(p. 8-9)**
Recruitment	15	Strategies for achieving adequate participant enrolment to reach target sample size **(p. 10)**

Methods: Assignment of interventions (for controlled trials)

Allocation:

For peer review only - http://bmjopen.bmj.com/site/about/guidelines.xhtml

Sequence generation	16a	Method of generating the allocation sequence (eg, computer-generated random numbers), and list of any factors for stratification. To reduce predictability of a random sequence, details of any planned restriction (eg, blocking) should be provided in a separate document that is unavailable to those who enrol participants or assign interventions **(p. 13)**
Allocation concealment mechanism	16b	Mechanism of implementing the allocation sequence (eg, central telephone; sequentially numbered, opaque, sealed envelopes), describing any steps to conceal the sequence until interventions are assigned **(p. 13)**
Implementation	16c	Who will generate the allocation sequence, who will enrol participants, and who will assign participants to interventions **(p. 13)**
Blinding (masking)	17a	Who will be blinded after assignment to interventions (eg, trial participants, care providers, outcome assessors, data analysts), and how **(p. 16-17)**
	17b	If blinded, circumstances under which unblinding is permissible, and procedure for revealing a participant's allocated intervention during the trial **(not applicable)**
**Methods: Data collection, management, and analysis**		
Data collection methods	18a	Plans for assessment and collection of outcome, baseline, and other trial data, including any related processes to promote data quality (eg, duplicate measurements, training of assessors) and a description of study instruments (eg, questionnaires, laboratory tests) along with their reliability and validity, if known. Reference to where data collection forms can be found, if not in the protocol **(p. 10-13)**
	18b	Plans to promote participant retention and complete follow-up, including list of any outcome data to be collected for participants who discontinue or deviate from intervention protocols **(p.14, p. 16, p. 17)**
Data management	19	Plans for data entry, coding, security, and storage, including any related processes to promote data quality (eg, double data entry; range checks for data values). Reference to where details of data management procedures can be found, if not in the protocol **(p. 17)**
Statistical methods	20a	Statistical methods for analysing primary and secondary outcomes. Reference to where other details of the statistical analysis plan can be found, if not in the protocol **(p. 13-14)**
	20b	Methods for any additional analyses (eg, subgroup and adjusted analyses) **(p. 14)**
	20c	Definition of analysis population relating to protocol non-adherence (eg, as randomised analysis), and any statistical methods to handle missing data (eg, multiple imputation) **(p. 14)**

For peer review only - http://bmjopen.bmj.com/site/about/guidelines.xhtml

Methods: Monitoring

Data monitoring	21a	Composition of data monitoring committee (DMC); summary of its role and reporting structure; statement of whether it is independent from the sponsor and competing interests; and reference to where further details about its charter can be found, if not in the protocol. Alternatively, an explanation of why a DMC is not needed **(p. 18-19)**
	21b	Description of any interim analyses and stopping guidelines, including who will have access to these interim results and make the final decision to terminate the trial **(p. 14)**
Harms	22	Plans for collecting, assessing, reporting, and managing solicited and spontaneously reported adverse events and other unintended effects of trial interventions or trial conduct **(p. 17)**
Auditing	23	Frequency and procedures for auditing trial conduct, if any, and whether the process will be independent from investigators and the sponsor **(p. 18-19**

Ethics and dissemination

Research ethics approval	24	Plans for seeking research ethics committee/institutional review board (REC/IRB) approval **(p. 17)**
Protocol amendments	25	Plans for communicating important protocol modifications (eg, changes to eligibility criteria, outcomes, analyses) to relevant parties (eg, investigators, REC/IRBs, trial participants, trial registries, journals, regulators) **(p. 17)**
Consent or assent	26a	Who will obtain informed consent or assent from potential trial participants or authorised surrogates, and how (see Item 32) **(p. 10)**
	26b	Additional consent provisions for collection and use of participant data and biological specimens in ancillary studies, if applicable **(non applicable)**
Confidentiality	27	How personal information about potential and enrolled participants will be collected, shared, and maintained in order to protect confidentiality before, during, and after the trial **(p. 18)**
Declaration of interests	28	Financial and other competing interests for principal investigators for the overall trial and each study site **(p. 18)**
Access to data	29	Statement of who will have access to the final trial dataset, and disclosure of contractual agreements that limit such access for investigators **(p. 19)**
Ancillary and post-trial care	30	Provisions, if any, for ancillary and post-trial care, and for compensation to those who suffer harm from trial participation **(p. 17)**

For peer review only

Dissemination policy	31a	Plans for investigators and sponsor to communicate trial results to participants, healthcare professionals, the public, and other relevant groups (eg, via publication, reporting in results databases, or other data sharing arrangements), including any publication restrictions **(p. 17)**
	31b	Authorship eligibility guidelines and any intended use of professional writers **(p. 17)**
	31c	Plans, if any, for granting public access to the full protocol, participant-level dataset, and statistical code **(p. 19)**
**Appendices**		
Informed consent materials	32	Model consent form and other related documentation given to participants and authorised surrogates **(supplementary materials)**
Biological specimens	33	Plans for collection, laboratory evaluation, and storage of biological specimens for genetic or molecular analysis in the current trial and for future use in ancillary studies, if applicable **(not applicable)**

*It is strongly recommended that this checklist be read in conjunction with the SPIRIT 2013 Explanation & Elaboration for important clarification on the items. Amendments to the protocol should be tracked and dated. The SPIRIT checklist is copyrighted by the SPIRIT Group under the Creative Commons "Attribution-NonCommercial-NoDerivs 3.0 Unported" license.

For peer review only

BMJ Open

Effect of goal-directed mobilization intervention compared with standard care on physical activity among medical inpatients: protocol for the GoMob-in randomized controlled trial

Journal:	*BMJ Open*
Manuscript ID	bmjopen-2021-058509.R1
Article Type:	Protocol
Date Submitted by the Author:	04-Apr-2022
Complete List of Authors:	Liechti, Fabian; University of Bern, Department of General Internal Medicine<br>Heinzmann, Jeannelle; University of Bern, Department of General Internal Medicine<br>Schmidt Leuenberger, Joachim; Inselspital University Hospital Bern, Department of Physiotherapy<br>Limacher, Andreas; Universitat Bern Institut fur Sozial- und Praventivmedizin<br>Wertli, Maria M; Inselspital Universitatsspital Bern, Department of General Internal Medicine<br>Verra, Martin; Inselspital University Hospital Bern, Department of Physiotherapy
**Primary Subject Heading**:	Geriatric medicine
Secondary Subject Heading:	Communication, Evidence based practice, General practice / Family practice, Patient-centred medicine, Rehabilitation medicine
Keywords:	Quality in health care < HEALTH SERVICES ADMINISTRATION & MANAGEMENT, INTERNAL MEDICINE, Rehabilitation medicine < INTERNAL MEDICINE, PREVENTIVE MEDICINE

SCHOLARONE™
Manuscripts

Effect of goal-directed mobilization intervention compared with standard care on physical activity among medical inpatients: protocol for the GoMob-in randomized controlled trial

Fabian D. Liechti[1], Jeannelle Heinzmann[1], Joachim M. Schmidt Leuenberger[2], Andreas Limacher[3], Maria M. Wertli[1], Martin L. Verra[2]

[1]Department of General Internal Medicine, Inselspital, Bern University Hospital, University of Bern, Bern, Switzerland

[2]Department of Physiotherapy, Insel Group, Bern University Hospital, Bern, Switzerland.

[3]CTU Bern, University of Bern, Bern, Switzerland

Corresponding author:

Fabian Liechti, MD, PhD

Department of General Internal Medicine

Bern University Hospital

Freiburgstrasse 8

3010 Bern

Mail: fabian.liechti@insel.ch

Phone: +41 31 632 21 11

For peer review only - http://bmjopen.bmj.com/site/about/guidelines.xhtml

Abstract

Introduction: Despite the fact that immobilization is a major contributor to morbidity and mortality, patients hospitalized in general internal medicine (GIM) wards spend up to 50% of time in bed. Previous studies in selected patient populations, showed increased mobility after implementation of goal-directed mobilization (GDM). Due to the study design used so far, the degree of evidence is generally low. The effect of GDM on clinical outcomes and economically relevant indicators in patients hospitalized in GIM wards is currently unknown. This study aims to evaluate a GDM intervention compared to standard care on physical activity (de Morton Mobility Index, DEMMI) in medical inpatients.

Methods and analysis: GoMob-in is a randomized, controlled, open-label study with blinded outcome assessment. We plan to enrol 160 inpatients with indication for physiotherapy on GIM wards of a tertiary hospital in Bern, Switzerland. Adult patients newly hospitalized on GIM wards will be included in the study. The primary outcome will be the change in the DEMMI score between baseline and 5 days. Secondary outcomes are change of DEMMI (inclusion to hospital discharge), mobilization time (inclusion to day 5, inclusion to discharge), in-hospital delirium episodes, number of in-hospital falls, length of stay, number of falls within 3 months, number of re-hospitalizations and all-cause mortality within 3 months, change in independence during activities of daily living, concerns of falling, and quality of life within 3 months, and destination after 3 months. Patients in the intervention group will be attributed a regularly updated individual mobility goal level made visible for all stakeholders and get a short educational intervention on GDM.

Ethics and Dissemination: This study has been approved by the responsible Ethics Board (Ethikkommission Bern / 2020-02305). Written informed consent will be obtained from participants before study inclusion. Results will be published in open access policy peer-reviewed journals.

Trial registration: ClinicalTrials.gov, NCT04760392.

For peer review only - http://bmjopen.bmj.com/site/about/guidelines.xhtml

Article Summary

Strengths and limitations of this study

- This is the first study evaluating goal-directed mobilisation in hospitalised general internal medicine patients using blinded outcome assessment.
- The primary endpoint physical activity will be assessed by change in the de Morton Mobility Index (DEMMI) score between baseline and day 5, a reliable tool without floor or ceiling effects.
- Activity will be recorded by an accelerometer to record the effect of the intervention.
- The study will also assess independence during activities of daily living, quality of life, number of re-hospitalizations, and all-cause mortality at 3 months follow-up.
- The study design is limited by the fact that blinding is not feasible for the interventions and some outcomes.

Keywords: mobilisation, goal-directed mobility, randomized controlled trial, physiotherapy, interprofessional, outcome

Word count: 3336 words.

Protocol Version 1.5, 27.08.2021

For peer review only

For peer review only - http://bmjopen.bmj.com/site/about/guidelines.xhtml

Introduction

Decreased mobility during hospitalization is associated with muscle loss, risk of falls, delirium, functional decline, and an increased mortality, especially in the elderly.[1-4] Bedrest of 10 days in healthy elderly resulted in a substantial loss of muscle strength, power, aerobic capacity, and a reduction in physical activity.[5] Evidence suggests that interventions that promote early mobility have the potential to decrease the risk of falls,[6 7] to reduce functional decline during hospitalization,[8] to shorten duration of delirium[9] and to reduce the median LOS and mortality.[9 10] Therefore, early mobilization is recommended in most patients to prevent functional decline and frailty.[11 12] Despite recommendations to mobilize patients early, a low level of physical activity is still common and many patients spend most of the time in bed during hospitalization, irrespective of patient age.[3 12-14] In a recent Dutch study, inpatients spent almost half of the time in bed and were active only 10% of the time.[15] Most studies assessing interventions on mobilization focused on selected patients such as older adults or those admitted to intensive care units. Further, most studies failed to monitor mobility or long-term functional outcomes or had a retrospective design.[2 12 16-18]

Implementation of early mobilization in general internal medicine (GIM) remains challenging and staff resources-demanding, because mobilization is usually supervised by health care professionals such as physiotherapists. Individual goal-achievement strategies have the potential to complement physiotherapy in rehabilitation.[12 19] Thereby, a mobility goal is generally defined depending on the individual potential of the patient and communicated to the interprofessional team caring for the patient. Goal-directed mobility (GDM) programs in GIM showed in temporal cohorts a reduction in length-of hospital-stay (LOS) and a decrease of falls.[20 21] Most evidence stems from studies which usually integrated several other organizational changes or were non-randomized trials, e.g., implementation of a mobility scale and corresponding mobility goal in hospital wards.[22] However, pre-post settings do not consider general trends, e.g. reduction in LOS and comparators may differ significantly. In one randomized controlled trial, a mobility program using goal-setting, reduced the decline in

For peer review only - http://bmjopen.bmj.com/site/about/guidelines.xhtml

activities of daily living during acute hospitalization.[7 23] However, it included only a small number of older veterans, was labour intense and lacked long-term follow-up.[7]

Due to the low number of studies of limited methodological quality, the level of evidence is low and the impact of GDM on clinical and economic outcomes in general internal medicine patients is widely unknown.[24]

Therefore, this study aims to evaluate a standardized GDM program in acutely hospitalized medical inpatients on functional capacity during hospitalization and up to 3 months after study inclusion. All patients will receive physiotherapy treatment as indicated by the physician in charge. Compared to a control group with standard care, patients in the intervention group will receive additional instructions for GDM, physiotherapists will work with goal-setting, and the goal will be indicated for all stakeholders at the bedside.

Methods and analysis

The methods reporting of this randomized trial will follow the recommendations of the SPIRIT statement (**Table 1**).[25]

Table 1: Trial registration data

**Data category**	**Information**
Primary registry and trial identifying number	ClinicalTrials.gov<br>NCT04760392
Date of registration in primary registry	February 18, 2021
Secondary numbers	SNCTP000004280, BASEC2020-02305
Source(s) of monetary or material support	Swiss Society of General Internal Medicine (SGAIM) Foundation, Bern, Switzerland<br>Fondation Sana, Bern, Switzerland
Primary sponsor	Swiss Society of General Internal Medicine (SGAIM) Foundation, Bern, Switzerland (grant number: not applicable)

For peer review only - http://bmjopen.bmj.com/site/about/guidelines.xhtml

For peer review only

Secondary sponsor	Fondation Sana, Bern, Switzerland (grant number GF 2021-0048)
Contact for public queries	FDL fabian.liechti@insel.ch
Contact for scientific queries	FDL<br>Department of General Internal Medicine, Inselspital, Bern University Hospital, Bern, Switzerland
Public title	Goal-directed mobilization of medical inpatients – a randomized, controlled trial (GoMob-in trial)
Scientific title	Goal-directed mobilization of medical inpatients – a randomized, controlled trial (GoMob-in trial)
Study setting	Number of study centre(s): 1<br>Type of study centre(s): University Hospital, Department of General Internal Medicine<br>Location, Country: Bern, Switzerland
Health condition(s) or problem(s) studied	Inpatient mobility
Intervention(s)	Intervention group: Goal-directed mobilisation
	Control group: Standard of care
Key inclusion and exclusion criteria	Ages eligible for study: 18 years or older<br>Sexes eligible for study: both<br>Accepts healthy volunteers: no
	Inclusion criteria: Acute hospitalization on the general internal medicine ward, indication for physiotherapy
	Exclusion criteria: Inability to follow study procedures and give informed consent themselves, expected hospital stay for <5 days, bedrest, injuries or relevant neurologic deficits one or both lower extremities directly impairing walking capacity, terminal illness
Study type	Interventional<br>Allocation: randomized intervention<br>Masking: single blind (primary outcome assessor)<br>Primary purpose: prevention

For peer review only

For peer review only - http://bmjopen.bmj.com/site/about/guidelines.xhtml

Date of first enrolment	14.09.2021
Target sample size	160
Recruitment status	Recruiting
Primary outcome	Change of the de Morton Mobility Index (DEMMI) score between baseline and day 5
Key secondary outcome	1. Change in DEMMI score between baseline and discharge.<br>2. Mobilization time measured by accelerometer between inclusion and day 5.<br>3. Mobilization time between inclusion and discharge.<br>4. Number of delirium episodes.<br>5. Number of in-hospital falls.<br>6. Length-of-hospital-stay.<br>7. Total number of falls (with / without injuries) within 3 months.<br>8. Number of re-hospitalizations and all-cause mortality within 3 months.<br>9. Independence during activities of daily living: change in Barthel index between study inclusion and 3 months.<br>10. Change in Falls Efficacy Scale - International (FES-I) between baseline and 3 months.<br>11. Quality of life: change in EuroQol (EQ-5D-5L) between study inclusion and 3 months.<br>12. Destination at 3 months after study inclusion (may include: death, acute care hospital, rehabilitation, home, nursing home, others)

Study setting

This is an investigator initiated trial. The funder has no role in the study design, the trial oversight, data collection, analysis of the study, and publication of the results. The Goal-directed Mobilization of Medical Inpatients (GoMob-in) trial is a pragmatic, randomized, controlled, open-label study with a 3-month follow-up and a blind outcome assessment, being conducted at the Department of General Internal Medicine, Inselspital, Bern University Hospital, Bern, Switzerland.

For peer review only - http://bmjopen.bmj.com/site/about/guidelines.xhtml

Objectives

To evaluate the effect of GDM compared to standard care in hospitalized patients in GIM on physical activity as assessed by the de Morton Mobility Index (DEMMI) score.

Hypothesis

GDM improves physical activity assessed by DEMMI compared to standard of care in hospitalized patients in GIM.

Eligibility criteria

Inclusion criteria:

Subjects fulfilling all of the following inclusion criteria are eligible for the study:

- Acute hospitalization on the GIM ward (elective or urgent admission, at latest on the second day after internal or external hospital admission)
- Age 18 years or older
- Indication for physiotherapy
- Informed consent as documented by signature (written informed consent)

Exclusion criteria:

The presence of any one of the following exclusion criteria will lead to exclusion of the subjects:

- Inability to follow study procedures, i.e., due to language problems (unable to read, speak or understand German), psychological disorders, severe dementia (defined as to levels 5 - 7 in the Global Deterioration Scale),[26] blindness, patients unable to provide informed consent themselves
- Expected hospital stay for <5 days
- Medically indicated bedrest for more than 24 h, e.g. after surgery
- Injuries or relevant neurologic deficits one or both lower extremities directly impairing walking capacity (e.g. fractures, hemiplegia, previous use of a wheelchair or bedriddenness)
- Terminal illness (i.e., end-of-life care, dying phase)

For peer review only - http://bmjopen.bmj.com/site/about/guidelines.xhtml

For peer review only

- Pregnancy or breast feeding
- Previous enrolment in this study
- A study participant in the same patient room
- Enrolment of the investigator, his/her family members, employees and other dependent persons

Estimated sample size and power

The sample size calculation was based on the primary outcome of a clinically meaningful change in the de Morton Mobility Index (DEMMI) score between baseline and 5 days follow-up. The DEMMI score ranges from 0 to 100 points with 0 indicating no physical activity and 100 indicating maximal physical activity. Based on previous studies on changes in DEMMI scores during short-term hospitalization of roughly 10 days,[27 28] we expect a difference in change between the two groups of 5 score points. In an observational study of participants with low and high activity, standard deviation (SD) for changes in the DEMMI score was 7.8 and 8.5 points, respectively (personal communication).[27] In a study on older adult inpatients the SD was between 12.5 and 15.4 points for absolute DEMMI scores at admission and discharge, respectively.[28] The SD for changes is typically smaller than for absolute values; we therefore expect a rather conservative an SD of about 10 points for the change in our study.

To detect a difference in change of 5 score points with a SD of 10 points, at a two-sided alpha level of 0.05 with a power of 0.8, a sample size of 2x 64 participants is required. Based on an estimated drop-out rate of 20%, we aim to include 160 patients into the study (80 in each group).

Primary outcome

The primary outcome will be the change in physical activity level measured by performance in the DEMMI score between baseline and 5 days follow-up.[29] Trained study personnel will evaluate physical activity by using DEMMI score at baseline before randomization, while a physiotherapist blinded to the group allocation will assess DEMMI score 5 days later.

Secondary outcome

For peer review only - http://bmjopen.bmj.com/site/about/guidelines.xhtml

1. Change in DEMMI score between baseline at study inclusion (at latest on the second day after hospital admission) and discharge. Patients hospitalized for more than 2 weeks will be assessed on day 14 after study inclusion instead of at hospital discharge.
2. Percentage of time moving between study inclusion and day 5. Activity will be measured using an accelerometer (GENEActiv, Activinsights, Kimbolton, Cambridgeshire, United Kingdom) validated to assess activity in the hospital setting.[13 14 30]
3. Percentage of mobilization time between baseline and discharge or at 14 days (whichever occurs first).
4. Number of in-hospital delirium episodes (follow-up max. 14 days).
5. Number of in-hospital falls (follow-up max. 14 days)
6. LOS defined as days between hospital admission and discharge.
7. Total number of falls (with / without injuries) within 3 months after study inclusion.
8. Number of re-hospitalizations and all-cause mortality within 3 months after study inclusion.
9. Change in independence during activities of daily living (Barthel index) between baseline and 3 months after study inclusion.[31 32]
10. Change in Falls Efficacy Scale - International (FES-I) between baseline and 3 months after study inclusion.[33 34]
11. Change in quality of life (EQ-5D-5L) (visual analogue scale [EQ-VAS] and mobility dimension) between baseline and 3 months after study inclusion.[35-37]
12. Discharge destination (death, acute care hospital, rehabilitation, home, nursing home)
13. Reaching the minimal clinically important difference (MCID) of a change in DEMMI score (9 points) between baseline and discharge.[29 38]

Study procedure

This study will generate evidence about a specific, easily implementable intervention using little resources to promote mobility and evaluate its impact on significant clinical outcomes of GIM inpatients (**Figure 1**). Study recruitment started on September 13, 2021. Study completion is expected by December 15, 2022.

For peer review only - http://bmjopen.bmj.com/site/about/guidelines.xhtml

Pre-screening and recruitment:

All patients hospitalised on the GIM department will be pre-screened during weekdays within the first two days of hospitalisation for study participation.

Baseline assessment:

Study personnel will obtain written informed consent (**supplementary file**) by the participant. After study inclusion, each patient receives a structured systematic assessment by the study coordinator including (**Table 2**):

- Socio-demographic and anthropometric data (e.g. age, gender, weight and height, zip code [rural versus urban living area], marital status, nurse visit at home [German: Spitex], insurance coverage [private or not], admission mode [elective or urgent], transfer from intensive care unit [ICU], hospitalization in the last 6 months), previous use of mobility aids.
- Comorbidities will be reported by the participant, using an established, systematic method as previously described.[39]
- Functional status using Barthel index. The Barthel index is one of the most widely used and reliable functional outcome measures and is measured through an established and validated questionnaire where higher scores on a scale between 0 and 100 indicate greater independence in activities of daily living.[31] It can be conducted by telephone and a validated German version is available.[32] If the patient is not able to answer the questions, the Barthel index will be administered by interviewing the ward nurse caring for the patient, a family member or any other person caring for the patient before hospitalization (e.g. home care nurse).
- Concerns of falling using FES-I. FES-I is a validated 16-item tool to evaluate concerns of falling, with 16 score points indicating no concerns of falling and 64 score points indicating high levels of concern.[33] It can be conducted by telephone and a validated German version is available.[34]
- Quality of life will be assessed with the EQ-5D-5L questionnaire. The EQ-5D-5L[35 36] is an established, standardized measure of quality of life consisting of a descriptive

For peer review only

system compromising the 5 dimensions mobility, self-care, usual activities, pain / discomfort, and anxiety / depression with five levels each (no problems, slight problems, moderate problems, severe problems, and extreme problems) and a visual analogue scale with 0 indicating the worst health and 100 indicating the best health.[40] It can be conducted by telephone interview and a validated German version is available.[37]

- Physical activity will be assessed by a trained study nurse using the DEMMI score. The German version of the DEMMI is a valid[41] and reliable[40] instrument to measure physical activity in older adults in the acute hospital setting and has no floor or ceiling effect.[29 38 42] Based on 15 items (0 to 19 points with Rasch conversion to a score ranging from 0 [indicating poor physical activity] to 100 [indicating a high level of independent physical activity]), the DEMMI includes evaluation of movement in bed (3 items), chair (3), static balance (4), walking (2), and dynamic balance (3). Acute older adult patients with a DEMMI score below 41 score points had significantly lower Barthel index (38% vs. 79%), used more mobility aids, were more bedridden, longer hospitalized, and were more often discharged to rehabilitation compared to those with a higher DEMMI score.[43] No association was found for gender, age, weight, body mass index, Charlson comorbidity index or handgrip strength.[43] Trained personnel is able to complete the DEMMI in no more than 10 minutes.

Table 2: Summary of study procedures based on Standard Protocol Items: Recommendations for Interventional Trials (SPIRIT) schedule

EHR, electronic health record.

	1st visit	2nd visit[1]	3rd visit	Phone call
**Time points** (days after randomization)	0	5	at discharge[2]	90

[1] Omitted if 3rd visit before day 6
[2] latest on day 14

For peer review only - http://bmjopen.bmj.com/site/about/guidelines.xhtml

Oral and written patient information	+			
Written consent	+			
Inclusion-/exclusion criteria	+			
Physical activity (De Morton Mobility Index)	+	+	+	
Randomization	+			
Demographics	+			
Installation of accelerometer	+			
Read-out of accelerometer			+	
Number of in-hospital falls			+ / daily	
Number of delirium episodes (EHR)			+	
Length of stay (EHR)			+	
Medical history (EHR)			+	
Destination (EHR)			+	+
Concerns of falling (Falls Efficacy Scale – International, FES-I)	+			+
Independence during activities of daily living (Barthel-Index)	+			+
Quality of life (EQ-5D)	+			+
Number of falls (with / without injuries)				+
Number of re-hospitalisations				+
Mortality				+

For peer review only

Mobility measurements

To objectively measure mobilization, patients will be equipped with a wrist-worn tri-axis accelerometer (GENEActiv, Activinsights, Kimbolton, Cambridgeshire, United Kingdom). This accelerometer has been validated and proven to reliably measure physical activity in hospitalized patients.[44 45] At inclusion, a study nurse will instruct the patients to wear the accelerometer as long as possible during hospitalization (day and night time) on either wrist side. The accelerometers will be collected before discharge or transfer to another hospital unit. The measurement frequency will be 50 Hz. Accelerometer data will be extracted and analysed using the GGIR package for R.[46] Time mobilized (total minutes per day) will be calculated from time "moving" as total of time "inactive" and "static", excluding time "not worn". Mean acceleration in miliG/vector will be indicated.

For peer review only - http://bmjopen.bmj.com/site/about/guidelines.xhtml

Randomization and allocation procedures

After patient registration in the data entry system (REDCap) and confirmation of all inclusion and exclusion criteria, patients are randomized 1:1 to one of the two study groups. Randomization will be blocked using varying block sizes of 2, 4, and 6. Moreover, randomization will be stratified according to the baseline DEMMI score (≤ 40 vs > 40 points) and age (< 65 vs ≥ 65 years). The allocation sequence (randomization list) will be generated by an independent data manager at CTU Bern, University of Bern, Bern, Switzerland not otherwise involved in the trial, and implemented in the data entry system to ensure concealment of allocation.

Statistical analysis

The statistical analysis will be performed by CTU Bern, University of Bern, using the statistical software packages Stata or R. All recorded and derived variables will be presented using descriptive summary tables. Continuous variables will be summarized by mean and SD or median and interquartile range (IQR) as appropriate. Categorical variables will be summarized with absolute and relative frequencies.

Primary analysis will be intention-to-treat. The primary outcome change in DEMMI score from randomization to day 5 will be compared between both intervention groups using a linear model. The model will be adjusted for the baseline DEMMI score and stratification factors used for randomization.

Secondary continuous outcomes will be analysed using the same approach as described above. To assess change in quality of life, we will analyse the difference between baseline and day 5 or day 14 in the visual analogue scale and the mobility dimension of the EQ-5D-5L. Count outcomes (number of delirium episodes, falls, and re-hospitalizations) will be assessed using a negative binomial model, categorical outcomes (destination) using a multinomial logistic model, binary outcomes (reaching MCID) using logistic regression, and time-to-event outcomes (LOS, mortality) using a Cox regression model. All models will be adjusted for stratification factors. In a per-protocol analysis, patients that violated any eligibility criteria, did not receive the allocated intervention, or were discharged before day 4 will be disregarded.

For peer review only - http://bmjopen.bmj.com/site/about/guidelines.xhtml

For peer review only

All effect measures will be accompanied by 95% confidence intervals (CI). The statistical testing will be two-sided with a type I error of 5%. The data analysts will be blinded to the group.

We will perform subgroup analysis for age groups <65 years vs. ≥65 years, initial performance in physical activity (DEMMI ≤40 vs. DEMMI >40), and pre-hospital mobility (no mobility aid vs. pre-hospital use of mobility aid) because previous studies showed that older age categories had significantly lower mean DEMMI scores[47], and lower Barthel index and use of mobility aids or being bedridden was associated with lower DEMMI scores.[43]

Any deviation from the original statistical plan will be described and justified in the final trial report. There is no interim analysis planned, i.e. there are no stopping rules on the individual or trial level.

Handling of missing data and drop-outs

If outcomes are missing, we will employ multiple imputation in the primary analysis and additionally perform an available case analysis as sensitivity analysis disregarding missing data. The DEMMI score that is measured two times during follow-up will additionally be evaluated in a repeated measures mixed-effects linear model, additionally introducing a random intercept for patients into the model.

Interventions

Experimental group: In patients assigned to GDM, a trained physiotherapist will provide a short educational intervention on GDM. Further, the physiotherapist will define a personal mobility goal based on an adapted version of the Johns Hopkins Highest Level of Mobility (JH-HLM) scale (**Table 3, Supplementary Figure 1**).[11 20] To avoid a ceiling effect in our population, compared to the original version of the JH-HLM, we added the following mobility goal: “Level 8: Walking 75 m or more (30 min or stairs) and no bed rest during daytime” and skipped the original “Level 1: Only lying”.

For peer review only - http://bmjopen.bmj.com/site/about/guidelines.xhtml

Table 3: Comparison of Johns Hopkins Highest Level of Mobility Scale (JH-HLM) and GoMob-in mobility goal levels

**Level**	**JH-HLM**	**GoMob-in mobility goal level**
**1**	only lying	bed activities
**2**	bed activities	sit at edge of bed
**3**	sit at edge of bed	transfer to chair/commode
**4**	transfer to chair/commode	standing for >1 minute
**5**	standing for >1 minute	walking 10+ steps
**6**	walking 10+ steps	walking 7.5 m or more
**7**	walking 7.5 m or more	walking 75 m or more
**8**	walking 75 m or more	Walking 75 m or more (30 min or stairs) and no bed rest during daytime

Participants will be instructed, that they should aim for successful completion of the task at least 3 times daily. The mobility goal level will be defined regularly by the treating physiotherapist together with the patient taking into account the current physical activity. Re-assessment will serve as opportunity for a short motivational intervention ("booster session"). The mobility goal level should be increased regularly. In patients reaching the highest mobility goal level, a note will be placed on the bed to indicate the patient to use the bed only for examinations and for sleep. All medical personnel (e.g., nurses) caring for the participant are allowed to adapt the mobility goal level. Increasing the mobility goal level more than one step or lowering it is also allowed. The study personnel will instruct nurses and physicians caring for participants in the intervention group regularly about the ongoing trial and its aims, i.e., responsible resident physicians will be informed by e-mail after randomization of a participant to the intervention group, nurses will be instructed orally and with leaflets at staff meetings at the beginning of the study and if deemed necessary.

The mobility goal level will be depicted on a patient board next to the bed, visible for all stakeholders (patients, visiting friends and family, physicians, nurses, physiotherapists).

For peer review only - http://bmjopen.bmj.com/site/about/guidelines.xhtml

Control group: Patients assigned to the control group, will receive standard treatment of the GIM wards. Mobility goals will not be routinely set nor encouraged interprofessionally. Patients will receive physiotherapist's guided training as prescribed by the resident in charge (resident physician / "Assistenzarzt"). As in any exercise trial, physiotherapists providing the standard treatment cannot be blinded to participation of the patients in the control group. They address the specific needs and provide general non-systematic information about the importance of mobilization during hospitalization as part of their routine instruction for all patients.

The adherence to intervention protocol will be assessed by presence of the patient board displaying the mobility goal level at discharge.

Follow-up

Nurses will record falls in the electronic health record (HER). Hospital parameters, e.g., discharge destination, end of hospitalization, LOS will be collected from the EHR at discharge. Medical data, e.g., number of falls and delirium episodes during hospitalisation, main diagnosis, and comorbidities will be systematically collected from the discharge letter.

Physical activity will be assessed using DEMMI in all participants on day 5 after study inclusion and on the day of discharge or day 14 (whichever occurs first).

Three months after study inclusion, all participants will be inquired by telephone interview for Barthel index, FES-I, EQ-5D, number of falls since study inclusion (with / without injuries), current destination, re-hospitalizations and all-cause mortality. If the patient cannot be reached, a family member or the treating general practitioner will be called to complement data. The patient will be asked to send the falls calendar to the study team. If discrepancy is noted compared to the phone interview, the participant will be called for validation.

Blinding

The DEMMI will be assessed by an independent physiotherapist not otherwise involved in the treatment of patients on the ward and to whom the group allocation of the participant is not declared. The patient will be instructed to not reveal the group allocation to the DEMMI assessor.

For peer review only

The statistician responsible for the final analysis will have no access to the group allocation until the primary analysis of the trial is finished. The data analysis will be conducted according to a pre-specified statistical analysis plan.

Data management

Study data will be collected and managed using REDCap electronic data capture tools hosted at CTU, University of Bern, Bern, Switzerland.[48 49] REDCap (Research Electronic Data Capture) is a secure, web-based software platform designed to support data capture for research studies, providing 1) an intuitive interface for validated data capture; 2) audit trails for tracking data manipulation and export procedures; 3) automated export procedures for seamless data downloads to common statistical packages; and 4) procedures for data integration and interoperability with external sources.

Safety

Protocol violations should not lead to treatment discontinuation unless they indicate a significant risk to patient safety. We will comply with all regulations concerning safety measures in clinical trials as set forth by the ethical committee. The investigators will report any serious adverse events occurring during clinical trials, independent of direct causal relationship with the intervention, within 24 hours.

Data monitoring committee

Data monitoring will be conducted by the CTU Bern, University of Bern, Bern, Switzerland and include routine site monitoring visits according to a specific monitoring plan. Data collection, handling of the data, and the analysis will be done by the researchers at the University of Bern, Bern, Switzerland.

Patient and public involvement

No patients were involved in the design of the study.

Ethics and dissemination

The trial will be performed in compliance with the Declaration of Helsinki and its amendments, and it has been approved by the responsible ethics board (Ethikkommission Bern, Bern,

For peer review only - http://bmjopen.bmj.com/site/about/guidelines.xhtml

Switzerland, 2020-02305). Important protocol modifications will be communicated to the Ethics Board and participating study sites. Potential participants must provide written informed consent before entering the study. Subjects can leave the study at any time for any reason without any negative consequences for their further treatment. When a patient ends the treatment phase of the study prematurely, we will record the date and reason for early treatment discontinuation. If possible, the end of treatment evaluations will be collected. Insurance coverage will be provided for all study participants by the study sponsor.

The results of the main trial and each of the secondary outcomes will be submitted for publication in open access peer-reviewed journals. The GoMob-in study group is an independent academic research group, which will not employ professional writers. Confidentiality of patients' data is ensured by all stakeholders at all times; patients will not be identified by names or identifiers such as birth date in any documents leaving the study site. Whenever data are exported from the database (e.g. for analysis), data will be coded.

For peer review only

Contributors

FDL designed the study. MW, AL, MLV, JS, and JH critically commented on the methods and contributed to the development of the study. FDL wrote the first draft of the manuscript. All authors revised the manuscript and approved the final version.

Funding

The GoMob-in trial is funded by the Swiss Society of General Internal Medicine (SGAIM) Foundation, Bern, Switzerland (grant number: not applicable) and the Fondation Sana, Bern, Switzerland (grant number: GF 2021-0048). The funders play no role in the data acquisition, data storage, and analysis.

Competing interests

The funder plays no role in the study design, collection of data, analysis, and writing of the manuscript. There is no conflict of interest regarding intellectual content and proprietary affairs.

For peer review only - http://bmjopen.bmj.com/site/about/guidelines.xhtml

The authors and investigators will have full access to the final trial dataset. No contractual agreements limit such access for investigators.

Data availability statement

Data will be available upon reasonable request.

Trial sponsor investigator

Fabian D. Liechti, MD, PhD

Department of General Internal Medicine

Bern University Hospital

Freiburgstrasse 8

3010 Bern

Mail: fabian.liechti@insel.ch

Phone: +41 31 632 21 11

For peer review only

References

1. Creditor MC. Hazards of hospitalization of the elderly. *Annals of internal medicine* 1993;118(3):219-23. doi: 10.7326/0003-4819-118-3-199302010-00011
2. Pashikanti L, Von Ah D. Impact of early mobilization protocol on the medical-surgical inpatient population: an integrated review of literature. *Clinical nurse specialist CNS* 2012;26(2):87-94. doi: 10.1097/NUR.0b013e31824590e6
3. Brown CJ, Friedkin RJ, Inouye SK. Prevalence and outcomes of low mobility in hospitalized older patients. *Journal of the American Geriatrics Society* 2004;52(8):1263-70. doi: 10.1111/j.1532-5415.2004.52354.x
4. Zisberg A, Shadmi E, Sinoff G, et al. Low mobility during hospitalization and functional decline in older adults. *Journal of the American Geriatrics Society* 2011;59(2):266-73. doi: 10.1111/j.1532-5415.2010.03276.x
5. Kortebein P, Symons TB, Ferrando A, et al. Functional impact of 10 days of bed rest in healthy older adults. *The journals of gerontology Series A, Biological sciences and medical sciences* 2008;63(10):1076-81. doi: 10.1093/gerona/63.10.1076
6. Growdon ME, Shorr RI, Inouye SK. The Tension Between Promoting Mobility and Preventing Falls in the Hospital. *JAMA internal medicine* 2017;177(6):759-60. doi: 10.1001/jamainternmed.2017.0840
7. Brown CJ, Foley KT, Lowman JD, Jr., et al. Comparison of Posthospitalization Function and Community Mobility in Hospital Mobility Program and Usual Care Patients: A Randomized Clinical Trial. *JAMA internal medicine* 2016;176(7):921-7. doi: 10.1001/jamainternmed.2016.1870
8. Cohen Y, Zisberg A, Chayat Y, et al. Walking for Better Outcomes and Recovery: The Effect of WALK-FOR in Preventing Hospital-Associated Functional Decline Among Older Adults. *The journals of gerontology Series A, Biological sciences and medical sciences* 2019;74(10):1664-70. doi: 10.1093/gerona/glz025
9. Hunter A, Johnson L, Coustasse A. Reduction of intensive care unit length of stay: the case of early mobilization. *The health care manager* 2014;33(2):128-35. doi: 10.1097/HCM.0000000000000006
10. Moore JE, Liu B, Khan S, et al. Can the effects of the mobilization of vulnerable elders in Ontario (MOVE ON) implementation be replicated in new settings: an interrupted time series design. *BMC geriatrics* 2019;19(1):99. doi: 10.1186/s12877-019-1124-0
11. Hoyer EH, Young DL, Klein LM, et al. Toward a Common Language for Measuring Patient Mobility in the Hospital: Reliability and Construct Validity of Interprofessional Mobility Measures. *Physical therapy* 2018;98(2):133-42. doi: 10.1093/ptj/pzx110
12. Smart DA, Dermody G, Coronado ME, et al. Mobility Programs for the Hospitalized Older Adult: A Scoping Review. *Gerontology & geriatric medicine* 2018;4:2333721418808146. doi: 10.1177/2333721418808146
13. Mudge AM, McRae P, McHugh K, et al. Poor mobility in hospitalized adults of all ages. *Journal of hospital medicine* 2016;11(4):289-91. doi: 10.1002/jhm.2536
14. Brown CJ, Redden DT, Flood KL, et al. The underrecognized epidemic of low mobility during hospitalization of older adults. *Journal of the American Geriatrics Society* 2009;57(9):1660-5. doi: 10.1111/j.1532-5415.2009.02393.x
15. Valkenet K, Bor P, van Delft L, et al. Measuring physical activity levels in hospitalized patients: a comparison between behavioural mapping and data from an accelerometer. *Clinical rehabilitation* 2019;33(7):1233-40. doi: 10.1177/0269215519836454
16. Liu B, Moore JE, Almaawiy U, et al. Outcomes of Mobilisation of Vulnerable Elders in Ontario (MOVE ON): a multisite interrupted time series evaluation of an

For peer review only - http://bmjopen.bmj.com/site/about/guidelines.xhtml

implementation intervention to increase patient mobilisation. *Age and ageing* 2018;47(1):112-19. doi: 10.1093/ageing/afx128

17. Hashem MD, Parker AM, Needham DM. Early Mobilization and Rehabilitation of Patients Who Are Critically Ill. *Chest* 2016;150(3):722-31. doi: 10.1016/j.chest.2016.03.003
18. Cameron S, Ball I, Cepinskas G, et al. Early mobilization in the critical care unit: A review of adult and pediatric literature. *Journal of critical care* 2015;30(4):664-72. doi: 10.1016/j.jcrc.2015.03.032
19. Hurn J, Kneebone I, Cropley M. Goal setting as an outcome measure: A systematic review. *Clinical rehabilitation* 2006;20(9):756-72. doi: 10.1177/0269215506070793
20. Hoyer EH, Friedman M, Lavezza A, et al. Promoting mobility and reducing length of stay in hospitalized general medicine patients: A quality-improvement project. *Journal of hospital medicine* 2016;11(5):341-7. doi: 10.1002/jhm.2546
21. Exum E, Hull BL. The Process of Implementing a Mobility Technician in the General Medicine and Surgical Population to Increase Patient Mobility and Improve Hospital Quality Measures: A Pilot Study. *Journal of Acute Care Physical Therapy* 2019;10(4) doi: 10.1097/JAT.0000000000000110
22. Klein LM, Young D, Feng D, et al. Increasing patient mobility through an individualized goal-centered hospital mobility program: A quasi-experimental quality improvement project. *Nurs Outlook* 2018;66(3):254-62. doi: 10.1016/j.outlook.2018.02.006 [published Online First: 2018/05/01]
23. Teodoro CR, Breault K, Garvey C, et al. STEP-UP: Study of the Effectiveness of a Patient Ambulation Protocol. *Medsurg nursing : official journal of the Academy of Medical-Surgical Nurses* 2016;25(2):111-6.
24. Cortes OL, Delgado S, Esparza M. Systematic review and meta-analysis of experimental studies: In-hospital mobilization for patients admitted for medical treatment. *Journal of advanced nursing* 2019;75(9):1823-37. doi: 10.1111/jan.13958
25. Chan AW, Tetzlaff JM, Gøtzsche PC, et al. SPIRIT 2013 explanation and elaboration: guidance for protocols of clinical trials. *BMJ (Clinical research ed)* 2013;346:e7586. doi: 10.1136/bmj.e7586 [published Online First: 2013/01/11]
26. Reisberg B, Ferris SH, de Leon MJ, et al. The Global Deterioration Scale for assessment of primary degenerative dementia. *Am J Psychiatry* 1982;139(9):1136-9. doi: 10.1176/ajp.139.9.1136
27. Karlsen A, Loeb MR, Andersen KB, et al. Improved Functional Performance in Geriatric Patients during Hospital Stay. *American Journal of Physical Medicine and Rehabilitation* 2017;96(5):e78-e84. doi: 10.1097/PHM.0000000000000671
28. Oestergaard AS, Mathiesen MH, Karlsen A, et al. In acutely admitted geriatric patients, offering increased physical activity during hospitalization decreases length of stay and can improve mobility. *Translational Sports Medicine* 2018;1(1):46-53. doi: 10.1002/tsm2.2
29. de Morton NA, Davidson M, Keating JL. The de Morton Mobility Index (DEMMI): an essential health index for an ageing world. *Health and quality of life outcomes* 2008;6:63. doi: 10.1186/1477-7525-6-63
30. Pedersen MM, Bodilsen AC, Petersen J, et al. Twenty-four-hour mobility during acute hospitalization in older medical patients. *The journals of gerontology Series A, Biological sciences and medical sciences* 2013;68(3):331-7. doi: 10.1093/gerona/gls165
31. Richards SH, Peters TJ, Coast J, et al. Inter-rater reliability of the Barthel ADL index: how does a researcher compare to a nurse? *Clinical rehabilitation* 2000;14(1):72-8. doi: 10.1191/026921500667059345

For peer review only

32. Lubke N, Meinck M, Von Renteln-Kruse W. [The Barthel Index in geriatrics. A context analysis for the Hamburg Classification Manual]. *Zeitschrift fur Gerontologie und Geriatrie* 2004;37(4):316-26. doi: 10.1007/s00391-004-0233-2
33. Yardley L, Beyer N, Hauer K, et al. Development and initial validation of the Falls Efficacy Scale-International (FES-I). *Age and ageing* 2005;34(6):614-9. doi: 10.1093/ageing/afi196
34. Dias N, Kempen GI, Todd CJ, et al. [The German version of the Falls Efficacy Scale-International Version (FES-I)]. *Zeitschrift fur Gerontologie und Geriatrie* 2006;39(4):297-300. doi: 10.1007/s00391-006-0400-8
35. EuroQol G. EuroQol--a new facility for the measurement of health-related quality of life. *Health policy* 1990;16(3):199-208. doi: 10.1016/0168-8510(90)90421-9
36. Foundation ER. EQ-5D-5L User Guide, Version 3.0 2019 [updated 09/2019. Available from: https://euroqol.org/publications/user-guides accessed 08.07.2020.
37. Ludwig K, Graf von der Schulenburg JM, Greiner W. German Value Set for the EQ-5D-5L. *PharmacoEconomics* 2018;36(6):663-74. doi: 10.1007/s40273-018-0615-8
38. de Morton NA, Davidson M, Keating JL. Validity, responsiveness and the minimal clinically important difference for the de Morton Mobility Index (DEMMI) in an older acute medical population. *BMC geriatrics* 2010;10:72. doi: 10.1186/1471-2318-10-72
39. Sangha O, Stucki G, Liang MH, et al. The Self-Administered Comorbidity Questionnaire: a new method to assess comorbidity for clinical and health services research. *Arthritis Rheum* 2003;49(2):156-63. doi: 10.1002/art.10993 [published Online First: 2003/04/11]
40. Dasenbrock L, Berg T, Lurz S, et al. [The De Morton Mobility Index for evaluation of early geriatric rehabilitation]. *Zeitschrift fur Gerontologie und Geriatrie* 2016;49(5):398-404. doi: 10.1007/s00391-016-1061-x
41. Braun T, Schulz RJ, Reinke J, et al. Reliability and validity of the German translation of the de Morton Mobility Index (DEMMI) performed by physiotherapists in patients admitted to a sub-acute inpatient geriatric rehabilitation hospital. *BMC geriatrics* 2015;15:58. doi: 10.1186/s12877-015-0035-y
42. de Morton NA, Davidson M, Keating JL. Reliability of the de Morton Mobility Index (DEMMI) in an older acute medical population. *Physiotherapy research international : the journal for researchers and clinicians in physical therapy* 2011;16(3):159-69. doi: 10.1002/pri.493
43. Melgaard D, Rodrigo-Domingo M, Morch MM, et al. DEMMI Scores, Length of Stay, and 30-Day Readmission of Acute Geriatric Patients in Denmark: A Cross-Sectional Observational Study with Longitudinal Follow-Up. *Geriatrics* 2019;4(1) doi: 10.3390/geriatrics4010008
44. Esliger DW, Rowlands AV, Hurst TL, et al. Validation of the GENEA Accelerometer. *Med Sci Sports Exerc* 2011;43(6):1085-93. doi: 10.1249/MSS.0b013e31820513be
45. Rowlands AV, Olds TS, Hillsdon M, et al. Assessing sedentary behavior with the GENEActiv: introducing the sedentary sphere. *Med Sci Sports Exerc* 2014;46(6):1235-47. doi: 10.1249/MSS.0000000000000224
46. Rowlands AV, Yates T, Davies M, et al. Raw Accelerometer Data Analysis with GGIR R-package: Does Accelerometer Brand Matter? *Med Sci Sports Exerc* 2016;48(10):1935-41. doi: 10.1249/MSS.0000000000000978

For peer review only - http://bmjopen.bmj.com/site/about/guidelines.xhtml

47. Macri EM, Lewis JA, Khan KM, et al. The de morton mobility index: normative data for a clinically useful mobility instrument. *Journal of aging research* 2012;2012:353252. doi: 10.1155/2012/353252
48. Harris PA, Taylor R, Thielke R, et al. Research electronic data capture (REDCap)--a metadata-driven methodology and workflow process for providing translational research informatics support. *J Biomed Inform* 2009;42(2):377-81. doi: 10.1016/j.jbi.2008.08.010 [published Online First: 2008/10/22]
49. Harris PA, Taylor R, Minor BL, et al. The REDCap consortium: Building an international community of software platform partners. *J Biomed Inform* 2019;95:103208. doi: 10.1016/j.jbi.2019.103208 [published Online First: 2019/05/13]

For peer review only

For peer review only - http://bmjopen.bmj.com/site/about/guidelines.xhtml

Figure Titles and legends

Figure 1: Study flowchart

DEMMI, de Morton Mobility Index; EHR, electronic health record; R, randomisation.

Supplementary Figure 1: GoMob-in mobility goal levels as attached to the patient's board close to its bed and icons used to indicate mobility restrictions

For peer review only

For peer review only - http://bmjopen.bmj.com/site/about/guidelines.xhtml

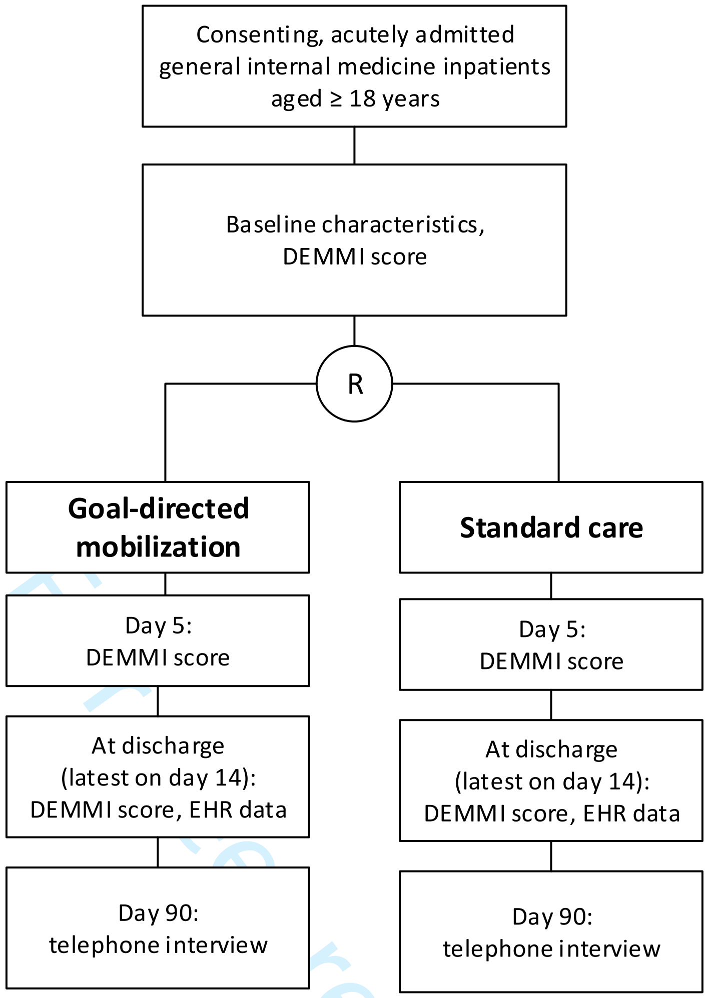
Consenting, acutely admitted general internal medicine inpatients aged ≥ 18 years
Baseline characteristics, DEMMI score
R
Goal-directed mobilization
Standard care
Day 5: DEMMI score
At discharge (latest on day 14): DEMMI score, EHR data
Day 90: telephone interview
Day 5: DEMMI score
At discharge (latest on day 14): DEMMI score, EHR data
Day 90: telephone interview

Stufe	Tages-Ziel (mindestens 3 x/Tag)
8	≥ 75 m gehen (30 min oder Treppe) & keine Bettruhe
7	≥ 75 m gehen
6	≥ 7,5 m gehen
5	≥ 10 Schritte gehen
4	≥ 1 Minute stehen
3	Transfer auf den Stuhl / Nachtstuhl
2	An die Bettkante sitzen
1	Aktivitäten im Bett / unselbständiger Transfer

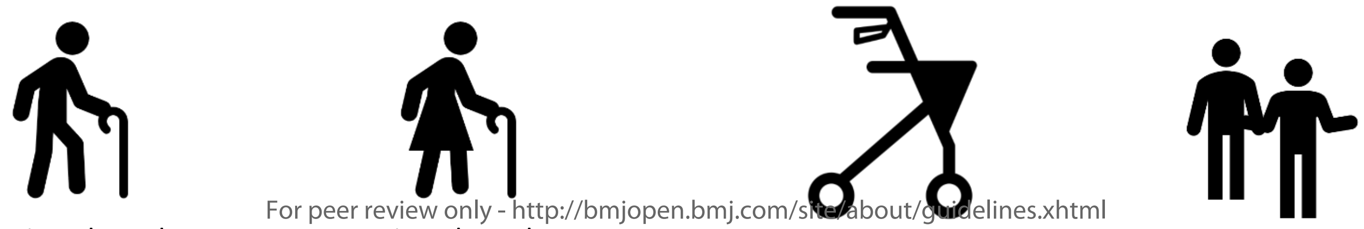

mit Gehstock   mit Gehstock   mit Rollator   in Begleitung

For peer review only - http://bmjopen.bmj.com/site/about/guidelines.xhtml

Klinik für Allgemeine Innere Medizin
Inselspital, 3010 Bern

Ziel-gerichtete Mobilisierung von Spitalpatienten (Goal-directed Mobilization of Medical inpatients – GoMob-in)

Diese Studie ist organisiert durch: Dr. med. Fabian D. Liechti, MD-PhD
Klinik für Allgemeine Innere Medizin KAIM
Inselspital / Universität Bern
Freiburgstrasse, 3010 Bern

Sehr geehrte Dame, sehr geehrter Herr,

Wir möchten Sie anfragen, ob Sie an einer klinischen Studie teilnehmen wollen. Im Folgenden wird Ihnen dieses Studienvorhaben dargestellt.

Detailliertere Information

1. Ziel der Studie

Wir möchten Sie hiermit bitten, an unserer klinischen Studie teilzunehmen. Die Studie untersucht, wie die Mobilität von Patienten im Spital verbessert werden könnte. Wir machen diese Studie, um herauszufinden, ob eine tägliche Zielvorgabe bei hospitalisierten Patienten Beweglichkeit, Lebensqualität und Überleben verbessert und Stürze, Verwirrtheit und Wiedereintritte ins Spital reduziert.

2. Auswahl

Es können alle Personen teilnehmen, die auf der Bettenstation der Allgemeinen Inneren Medizin des Inselspitals hospitalisiert werden (Eintritt vor weniger als zwei Tagen) und Physiotherapie durch die Stationsärztin oder den Stationsarzt verordnet erhalten haben. Ausserdem müssen Sie diese Einverständniserklärung verstehen und unterschreiben können. Nicht teilnehmen dürfen Personen welche voraussichtlich weniger als 5 Tage hospitalisiert werden, eine relevante Geheinschränkung (wie z. B. einen Beinbruch oder einen Schlaganfall mit Halbseitenlähmung) haben, vom Arzt Bettruhe verordnet bekommen oder schwanger/stillend sind.

3. Allgemeine Informationen

Viele Patienten, welche auf einer Bettenstation hospitalisiert werden, bewegen sich während des Spitalaufenthaltes wenig. Es ist bekannt, dass dieser Bewegungsmangel zu Komplikationen wie Muskelabbau, Stürzen, Verwirrtheit und verschlechterter Bewegungsfähigkeit führen kann. Deswegen wird empfohlen, dass sich die Patienten während der Hospitalisation viel bewegen – abhängig vom Gesundheitszustand und allenfalls durch den behandelnden Arzt verfügter Einschränkung.

Diese Studie wird an der Klinik für Allgemeine Innere Medizin am Inselspital Bern durchgeführt. Wir planen 160 Personen in die Studie einzuschliessen. In dieser Studie wollen wir klären, ob eine klare Kommunikation und Angabe eines individuellen Tagesziels die Beweglichkeit von hospitalisierten Personen verbessern kann.

Die Teilnehmenden werden zufällig einer von 2 Gruppen zugeordnet: einer Motivations-Gruppe und einer Kontroll-Gruppe. Die Wahrscheinlichkeit der Zuteilung auf eine Gruppe beträgt 50%. Zur Messung der Bewegung, wird Ihnen während des Spitalaufenthaltes ein Schrittzähler angehängt. Dieser misst Ihre Bewegung, woraus ein Computerprogramm berechnen kann, ob sie schlafen, stehen, sitzen oder gehen. In der Motivations-Gruppe wird den Patienten jeden Tag vorgegeben,

wie viel sie sich mindestens bewegen sollen. In der Kontroll-Gruppe bewegen sich die Patienten unabhängig von einem Tagesziel.

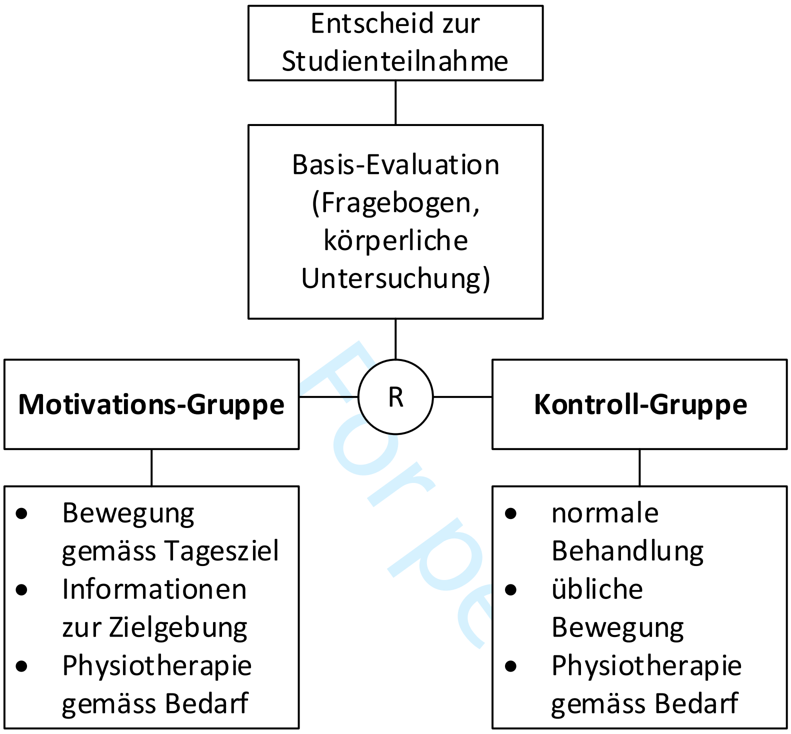

Abbildung 1: Übersicht der beiden Gruppen. (R = zufällige Zuordnung zu einer der beiden Gruppen)

Die aktive Teilnahme dauert solange wie der Spitalaufenthalt, jedoch höchstens 14 Tage und wird mit einem Fragebogen welcher nach 3 Monaten telefonisch beantwortet wird, abgeschlossen.

Wir machen diese Studie so, wie es die Gesetze in der Schweiz vorschreiben. Ausserdem beachten wir alle international anerkannten Richtlinien. Die zuständige Kantonale Ethikkommission hat die Studie geprüft und bewilligt.

Eine Beschreibung dieser Studie finden Sie auch auf der Internetseite des Bundesamtes für Gesundheit: www.kofam.ch

4. Ablauf

Nach Einwilligung zur Teilnahme an der Studie wird eine Eintrittsbefragung und -Untersuchung durchgeführt. Diese beinhaltet folgendes:

- Überprüfung der Ein- und Ausschlusskriterien der Studie
- Fragen zu Ihrer Person wie Grösse, Gewicht, Wohnort, Gehhilfen, Vorerkrankungen, etc.
- Fragebogen zur Gangunsicherheit, Selbständigkeit und Lebensqualität (ca. 30 min)
- Körperliche Untersuchung Ihrer Beweglichkeit (ca. 10-15 min)

Für die Studie sind keine zusätzlichen Blutentnahmen oder Röntgenuntersuchungen notwendig. Bei Frauen im gebährfähigen Alter wird aus einer bereits vorhandenen Blutprobe das Schwangerschaftshormon analysiert um eine Schwangerschaft auszuschliessen.

Die Untersuchung der Beweglichkeit wird am fünften Tag und vor Spitalaustritt oder spätestens nach 14 Tagen wiederholt. Sie müssen also für die Studie nicht länger im Spital bleiben als sonst vorgesehen.
Angaben zu ihrer Gesundheit wie z. B. Diagnosen, eingenommene Medikamente, Stürze werden wir zudem nach Ihrem Austritt aus Ihrer elektronischen Krankenakte entnehmen.

For peer review only - http://bmjopen.bmj.com/site/about/guidelines.xhtml

Wir werden ihnen ein Sturztagebuch mit nach Hause geben, wo sie allfällige Stürze dokumentieren können. Nach 3 Monaten werden wir Sie telefonisch kontaktieren um einige Fragen zu Ihrer Gesundheit zu stellen (Fragebogen zur Lebensqualität, zur Gangunsicherheit, zur Selbständigkeit, zum aktuellen Aufenthaltsort u. ä.). Sollten wir Sie wiederholt nicht erreichen, werden wir Ihre Angehörigen oder Ihren Hausarzt kontaktieren, um diese Fragen klären zu können.

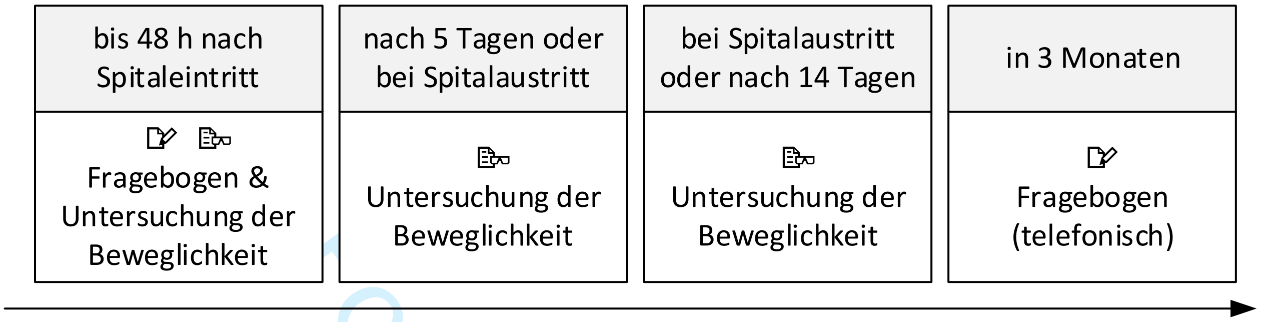

Abbildung 2: Ablauf der Untersuchungen während der Studie

Es kann sein, dass wir Sie von der Studie vorzeitig ausschliessen müssen. Das kann geschehen, wenn die Studie vorzeitig abgebrochen wird oder wir bei Ihnen ein Ausschlusskriterium finden, welches bei Einschluss nicht bekannt war.

5. Nutzen

Die Resultate der Studie können wichtig sein zur Erforschung der Mobilität bei Spitalpatienten.

6. Rechte

Sie nehmen freiwillig teil. Wenn Sie nicht mitmachen oder später Ihre Teilnahme zurückziehen wollen, müssen Sie dies nicht begründen. Ihre medizinische Behandlung/Betreuung ist unabhängig von Ihrem Entscheid gewährleistet. Sie dürfen jederzeit Fragen zur Studienteilnahme stellen. Wenden Sie sich dazu bitte an die Person, die am Ende dieser Information genannt ist.

7. Pflichten

Als Teilnehmer ist es notwendig, dass Sie

- sich an die notwendigen Vorgaben und Anforderungen der Studie durch den Prüfplan halten,
- Ihren Prüfarzt über den Verlauf der Erkrankung informieren und neue Symptome, neue Beschwerden und Änderungen im Befinden melden.

8. Risiken und Belastungen für die Teilnehmenden

Es sind keine Risiken der Studienintervention zu erwarten.

9. Andere Behandlungsmöglichkeiten

Sie müssen bei dieser Studie nicht teilnehmen. Wenn Sie nicht mitmachen, können Sie z. B. selbst auf eine ausreichende Bewegung während des Spitalaufenthalts achten.

10. Ergebnisse aus der Studie

Der Prüfarzt wird Sie während der Studie über alle neuen Erkenntnisse informieren, die den Nutzen der Studie oder Ihre Sicherheit und somit Ihr Einwilligung zur Teilnahme an der Studie beeinflussen können. Sie werden die Information mündlich und schriftlich erhalten.

11. Vertraulichkeit der Daten

Für diese Studie werden Ihre persönlichen und medizinischen Daten erfasst. Nur sehr wenige Fachpersonen werden Ihre unverschlüsselten Daten sehen, und zwar ausschliesslich, um Aufgaben im Rahmen der Studie zu erfüllen. Bei der Datenerhebung zu Studienzwecken werden die Daten verschlüsselt. Verschlüsselung bedeutet, dass alle Bezugsdaten, die Sie identifizieren

For peer review only - http://bmjopen.bmj.com/site/about/guidelines.xhtml

könnten (Name, Geburtsdatum), gelöscht und durch einen Schlüssel ersetzt werden. Die Schlüssel-Liste bleibt immer im Inselspital. Diejenigen Personen, die den Schlüssel nicht kennen, können daher keine Rückschlüsse auf Ihre Person ziehen. Bei einer Publikation sind die zusammengefassten Daten daher auch nicht auf Sie als Einzelperson rückverfolgbar. Ihr Name taucht niemals im Internet oder einer Publikation auf. Manchmal gibt es die Vorgabe zur Publikation bei einer Zeitschrift, dass Einzel-Daten (sogenannte Roh-Daten) übermittelt werden müssen. Wenn Einzel-Daten übermittelt werden müssen, dann sind die Daten immer verschlüsselt und somit ebenfalls nicht zu Ihnen als Person rückverfolgbar. Alle Personen, die im Rahmen der Studie Einsicht in Ihre Daten haben, unterliegen der Schweigepflicht. Die Vorgaben des Datenschutzes werden eingehalten und Sie als teilnehmende Person haben jederzeit das Recht auf Einsicht in Ihre Daten.
Möglicherweise wird diese Studie durch die zuständige Ethikkommission überprüft. Der Prüfarzt muss eventuell Ihre persönlichen und medizinischen Daten für solche Kontrollen offenlegen. Alle Personen müssen absolute Vertraulichkeit wahren.
Es ist möglich, dass Ihr nachbehandelnder Arzt kontaktiert wird, um Auskunft über Ihren Gesundheitszustand zu geben.

12. Rücktritt
Sie können jederzeit aufhören und von der Studie zurücktreten, wenn Sie das wünschen. Die bis dahin erhobenen Daten werden noch verschlüsselt ausgewertet, weil das ganze Projekt sonst seinen Wert verliert.
Nach der Auswertung werden Ihre Daten vollständig anonymisiert, d.h. Ihre Schlüsselzuordnung wird vernichtet, so dass danach niemand mehr erfahren kann, dass die Daten ursprünglich von Ihnen stammten.

13. Entschädigung für Teilnehmende
Wenn Sie an dieser Studie teilnehmen, bekommen Sie dafür keine Entschädigung. Es entstehen Ihnen oder Ihrer Krankenkasse keine Kosten durch die Teilnahme.

14. Haftung
Die Institution, die die Studie veranlasst hat und für die Durchführung verantwortlich ist, haftet für Schäden, welche Ihnen im Zusammenhang mit den Forschungshandlungen (z.B. Untersuchungen) entstehen könnten. Die Voraussetzungen und das Vorgehen dazu sind gesetzlich geregelt.
Falls Sie einen Schaden erlitten haben, so wenden Sie sich bitte an den Prüfarzt.

15. Finanzierung der Studie
Die Studie wird grösstenteils von der SGAIM Foundation (Stiftung der Schweizerischen Gesellschaft für Allgemeine Innere Medizin) bezahlt.

16. Kontaktperson(en)
Bei Fragen, Unsicherheiten oder Notfällen, die während der Studie oder danach auftreten, können Sie sich jederzeit an eine dieser Kontaktpersonen wenden.

Prüfarzt: Dr. med. Fabian D. Liechti, MD-PhD
Klinik für Allgemeine Innere Medizin KAIM
Inselspital / Universität Bern
Freiburgstrasse, 3010 Bern
fabian.liechti@insel.ch
Tel. +41 31 632 21 11 / Sucher: 181-7253

For peer review only - http://bmjopen.bmj.com/site/about/guidelines.xhtml

Klinik für Allgemeine Innere Medizin
Inselspital, 3010 Bern

Einwilligungserklärung

Schriftliche Einwilligungserklärung zur Teilnahme an einem Studienprojekt
Bitte lesen Sie dieses Formular sorgfältig durch. Bitte fragen Sie, wenn Sie etwas nicht verstehen oder wissen möchten. Für die Teilnahme ist Ihre schriftliche Einwilligung notwendig.

**BASEC-Nummer (nach Einreichung):**	
**Titel der Studie (wissenschaftlich und Laiensprache)**:	Ziel-gerichtete Mobilisierung von Spitalpatienten (Goal-directed Mobilization of Medical inpatients – GoMob-in)
**verantwortliche Institution (Sponsor mit Adresse)**:	Inselspital, Klinik für Allgemeine Innere Medizin, Freiburgstrasse, 3010 Bern
**Ort der Durchführung**:	Bern
**Verantwortlicher Prüfarzt / Studienkoordinator am Studienort:** Name und Vorname in Druckbuchstaben:	Dr. med. Fabian D. Liechti
**Teilnehmerin/Teilnehmer:** Name und Vorname in Druckbuchstaben: Geburtsdatum:	☐ weiblich ☐ männlich

- Ich wurde vom unterzeichnenden Prüfarzt mündlich und schriftlich über den Zweck, den Ablauf der Studie, über mögliche Vor- und Nachteile sowie über eventuelle Risiken informiert.
- Ich nehme an dieser Studie freiwillig teil und akzeptiere den Inhalt der abgegebenen schriftlichen Information. Ich hatte genügend Zeit, meine Entscheidung zu treffen.
- Ich wurde über mögliche andere Behandlungen und Behandlungsverfahren aufgeklärt.
- Meine Fragen im Zusammenhang mit der Teilnahme an dieser Studie sind mir beantwortet worden. Ich behalte die schriftliche Information und erhalte eine Kopie meiner schriftlichen Einwilligungserklärung.
- Ich bin mir bewusst, dass die in der Teilnehmerinformation genannten Pflichten einzuhalten sind. Im Interesse meiner Gesundheit kann mich der Prüfarzt jederzeit von der Studie ausschliessen.
- Ich bin einverstanden, dass mein Hausarzt über meine Teilnahme an der Studie informiert wird.
- Ich bin einverstanden, dass die zuständigen Fachleute des Sponsors, der zuständigen Ethikkommission zu Prüf- und Kontrollzwecken in meine unverschlüsselten Daten Einsicht nehmen dürfen, jedoch unter strikter Einhaltung der Vertraulichkeit.
- Bei Studienergebnissen, die direkt meine Gesundheit betreffen, werde ich informiert. Wenn ich das nicht wünsche, informiere ich meinen Prüfarzt.
- Ich weiss, dass meine gesundheitsbezogenen und persönlichen Daten nur in verschlüsselter Form zu Forschungszwecken für diese Studie weitergegeben werden können.
- Ich kann jederzeit und ohne Angabe von Gründen von der Studienteilnahme zurücktreten. Meine weitere medizinische Behandlung ist unabhängig von der Studienteilnahme immer gewährleistet. Die bis zum Rücktritt erhobenen Daten werden für die Auswertung zur Studie verwendet.
- Die Haftpflichtversicherung des Spitals/der Institution kommt für allfällige Schäden auf.

For peer review only

Klinik für Allgemeine Innere Medizin
Inselspital, 3010 Bern

Ort, Datum	Unterschrift Teilnehmerin/Teilnehmer

Bestätigung des Prüfarztes/der Prüfperson: Hiermit bestätige ich, dass ich dieser Teilnehmerin/ diesem Teilnehmer Wesen, Bedeutung und Tragweite der Studie erläutert habe. Ich versichere, alle im Zusammenhang mit dieser Studie stehenden Verpflichtungen gemäss dem geltenden Recht zu erfüllen. Sollte ich zu irgendeinem Zeitpunkt während der Durchführung der Studie von Aspekten erfahren, welche die Bereitschaft der Teilnehmerin/ des Teilnehmers zur Teilnahme an der Studie beeinflussen könnten, werde ich sie/ ihn umgehend darüber informieren.

Ort, Datum	Name und Vorname der Prüfärztin / des Prüfarztes / des Studienkoordinators / der Studienkoordinatorin in Druckbuchstaben<br><br>Unterschrift der Prüfärztin/des Prüfarztes des Studienkoordinators / der Studienkoordinatorin

For peer review only

For peer review only - http://bmjopen.bmj.com/site/about/guidelines.xhtml

SPIRIT

STANDARD PROTOCOL ITEMS: RECOMMENDATIONS FOR INTERVENTIONAL TRIALS

SPIRIT 2013 Checklist: Recommended items to address in a clinical trial protocol and related documents*

Section/item	Item No	Description
**Administrative information**		
Title	1	Descriptive title identifying the study design, population, interventions, and, if applicable, trial acronym **(p. 1)**
Trial registration	2a	Trial identifier and registry name. If not yet registered, name of intended registry **(p. 5)**
	2b	All items from the World Health Organization Trial Registration Data Set **(p. 5-7)**
Protocol version	3	Date and version identifier **(p. 3)**
Funding	4	Sources and types of financial, material, and other support **(p. 19)**
Roles and responsibilities	5a	Names, affiliations, and roles of protocol contributors **(p. 19)**
	5b	Name and contact information for the trial sponsor **(p. 19)**
	5c	Role of study sponsor and funders, if any, in study design; collection, management, analysis, and interpretation of data; writing of the report; and the decision to submit the report for publication, including whether they will have ultimate authority over any of these activities **(p. 19)**
	5d	Composition, roles, and responsibilities of the coordinating centre, steering committee, endpoint adjudication committee, data management team, and other individuals or groups overseeing the trial, if applicable (see Item 21a for data monitoring committee) **(p. 19)**
**Introduction**		
Background and rationale	6a	Description of research question and justification for undertaking the trial, including summary of relevant studies (published and unpublished) examining benefits and harms for each intervention **(p. 4)**
	6b	Explanation for choice of comparators **(p. 4-5)**
Objectives	7	Specific objectives or hypotheses **(p. 7)**

For peer review only - http://bmjopen.bmj.com/site/about/guidelines.xhtml

Trial design	8	Description of trial design including type of trial (eg, parallel group, crossover, factorial, single group), allocation ratio, and framework (eg, superiority, equivalence, noninferiority, exploratory) **(p. 7, 13)**

Methods: Participants, interventions, and outcomes

Study setting	9	Description of study settings (eg, community clinic, academic hospital) and list of countries where data will be collected. Reference to where list of study sites can be obtained **(p. 7)**
Eligibility criteria	10	Inclusion and exclusion criteria for participants. If applicable, eligibility criteria for study centres and individuals who will perform the interventions (eg, surgeons, psychotherapists) **(p. 7-8)**
Interventions	11a	Interventions for each group with sufficient detail to allow replication, including how and when they will be administered **(p. 14-16)**
	11b	Criteria for discontinuing or modifying allocated interventions for a given trial participant (eg, drug dose change in response to harms, participant request, or improving/worsening disease) **(p. 17)**
	11c	Strategies to improve adherence to intervention protocols, and any procedures for monitoring adherence (eg, drug tablet return, laboratory tests) **(p. 16)**
	11d	Relevant concomitant care and interventions that are permitted or prohibited during the trial **(p. 16)**
Outcomes	12	Primary, secondary, and other outcomes, including the specific measurement variable (eg, systolic blood pressure), analysis metric (eg, change from baseline, final value, time to event), method of aggregation (eg, median, proportion), and time point for each outcome. Explanation of the clinical relevance of chosen efficacy and harm outcomes is strongly recommended **(p. 8-9)**
Participant timeline	13	Time schedule of enrolment, interventions (including any run-ins and washouts), assessments, and visits for participants. A schematic diagram is highly recommended (see Figure) **(p. 12)**
Sample size	14	Estimated number of participants needed to achieve study objectives and how it was determined, including clinical and statistical assumptions supporting any sample size calculations **(p. 8-9)**
Recruitment	15	Strategies for achieving adequate participant enrolment to reach target sample size **(p. 10)**

Methods: Assignment of interventions (for controlled trials)

Allocation:

For peer review only - http://bmjopen.bmj.com/site/about/guidelines.xhtml

Sequence generation	16a	Method of generating the allocation sequence (eg, computer-generated random numbers), and list of any factors for stratification. To reduce predictability of a random sequence, details of any planned restriction (eg, blocking) should be provided in a separate document that is unavailable to those who enrol participants or assign interventions **(p. 13)**
Allocation concealment mechanism	16b	Mechanism of implementing the allocation sequence (eg, central telephone; sequentially numbered, opaque, sealed envelopes), describing any steps to conceal the sequence until interventions are assigned **(p. 13)**
Implementation	16c	Who will generate the allocation sequence, who will enrol participants, and who will assign participants to interventions **(p. 13)**
Blinding (masking)	17a	Who will be blinded after assignment to interventions (eg, trial participants, care providers, outcome assessors, data analysts), and how **(p. 16-17)**
	17b	If blinded, circumstances under which unblinding is permissible, and procedure for revealing a participant's allocated intervention during the trial **(not applicable)**
**Methods: Data collection, management, and analysis**		
Data collection methods	18a	Plans for assessment and collection of outcome, baseline, and other trial data, including any related processes to promote data quality (eg, duplicate measurements, training of assessors) and a description of study instruments (eg, questionnaires, laboratory tests) along with their reliability and validity, if known. Reference to where data collection forms can be found, if not in the protocol **(p. 10-13)**
	18b	Plans to promote participant retention and complete follow-up, including list of any outcome data to be collected for participants who discontinue or deviate from intervention protocols **(p.14, p. 16, p. 17)**
Data management	19	Plans for data entry, coding, security, and storage, including any related processes to promote data quality (eg, double data entry; range checks for data values). Reference to where details of data management procedures can be found, if not in the protocol **(p. 17)**
Statistical methods	20a	Statistical methods for analysing primary and secondary outcomes. Reference to where other details of the statistical analysis plan can be found, if not in the protocol **(p. 13-14)**
	20b	Methods for any additional analyses (eg, subgroup and adjusted analyses) **(p. 14)**
	20c	Definition of analysis population relating to protocol non-adherence (eg, as randomised analysis), and any statistical methods to handle missing data (eg, multiple imputation) **(p. 14)**

For peer review only - http://bmjopen.bmj.com/site/about/guidelines.xhtml

Methods: Monitoring

Data monitoring	21a	Composition of data monitoring committee (DMC); summary of its role and reporting structure; statement of whether it is independent from the sponsor and competing interests; and reference to where further details about its charter can be found, if not in the protocol. Alternatively, an explanation of why a DMC is not needed **(p. 18-19)**
	21b	Description of any interim analyses and stopping guidelines, including who will have access to these interim results and make the final decision to terminate the trial **(p. 14)**
Harms	22	Plans for collecting, assessing, reporting, and managing solicited and spontaneously reported adverse events and other unintended effects of trial interventions or trial conduct **(p. 17)**
Auditing	23	Frequency and procedures for auditing trial conduct, if any, and whether the process will be independent from investigators and the sponsor **(p. 18-19**

Ethics and dissemination

Research ethics approval	24	Plans for seeking research ethics committee/institutional review board (REC/IRB) approval **(p. 17)**
Protocol amendments	25	Plans for communicating important protocol modifications (eg, changes to eligibility criteria, outcomes, analyses) to relevant parties (eg, investigators, REC/IRBs, trial participants, trial registries, journals, regulators) **(p. 17)**
Consent or assent	26a	Who will obtain informed consent or assent from potential trial participants or authorised surrogates, and how (see Item 32) **(p. 10)**
	26b	Additional consent provisions for collection and use of participant data and biological specimens in ancillary studies, if applicable **(non applicable)**
Confidentiality	27	How personal information about potential and enrolled participants will be collected, shared, and maintained in order to protect confidentiality before, during, and after the trial **(p. 18)**
Declaration of interests	28	Financial and other competing interests for principal investigators for the overall trial and each study site **(p. 18)**
Access to data	29	Statement of who will have access to the final trial dataset, and disclosure of contractual agreements that limit such access for investigators **(p. 19)**
Ancillary and post-trial care	30	Provisions, if any, for ancillary and post-trial care, and for compensation to those who suffer harm from trial participation **(p. 17)**

For peer review only

Dissemination policy	31a	Plans for investigators and sponsor to communicate trial results to participants, healthcare professionals, the public, and other relevant groups (eg, via publication, reporting in results databases, or other data sharing arrangements), including any publication restrictions **(p. 17)**
	31b	Authorship eligibility guidelines and any intended use of professional writers **(p. 17)**
	31c	Plans, if any, for granting public access to the full protocol, participant-level dataset, and statistical code **(p. 19)**
**Appendices**		
Informed consent materials	32	Model consent form and other related documentation given to participants and authorised surrogates **(supplementary materials)**
Biological specimens	33	Plans for collection, laboratory evaluation, and storage of biological specimens for genetic or molecular analysis in the current trial and for future use in ancillary studies, if applicable **(not applicable)**

*It is strongly recommended that this checklist be read in conjunction with the SPIRIT 2013 Explanation & Elaboration for important clarification on the items. Amendments to the protocol should be tracked and dated. The SPIRIT checklist is copyrighted by the SPIRIT Group under the Creative Commons "Attribution-NonCommercial-NoDerivs 3.0 Unported" license.

For peer review only
